# A tumor-targeting black phosphorus-based nanoplatform for controlled chemo-photothermal therapy of breast cancer

**DOI:** 10.1016/j.mtbio.2025.101563

**Published:** 2025-02-07

**Authors:** Lin Yang, Ying Zhang, Jing Liu, Xiaofen Wang, Li Zhang, Hao Wan

**Affiliations:** a State Key Laboratory of Food Science and Resources, Nanchang University, Nanchang, 330047, PR China; b College of Pharmacy, Dalian Medical University, Dalian, 116044, PR China; c Cancer Research Center, Jiangxi University of Chinese Medicine, Nanchang, 330004, PR China

**Keywords:** Chemo-photothermal therapy, Controlled release, Tumor targeting, Indole-3-carbinol, Nano-sized black phosphorus

## Abstract

Combination therapy with high efficacy and precision shows great potential in breast cancer treatment. Herein, we developed a multifunctional nanocarrier (NBP@mSiO_2_-PEG-cRGD) for tumor-targeting chemo-photothermal therapy of breast cancer in a controlled manner. The nanocarrier was constructed by enveloping nano-sized black phosphorus (NBP) within a mesoporous silica shell (mSiO_2_) modified with the tumor-targeting peptide c(Arg-Gly-Asp-dPhe-Cys) (cRGD). Due to the existence of pore channels within mSiO_2_, NBP@mSiO_2_-PEG-cRGD achieved high loading efficiency of indole-3-carbinol (I3C) molecules (NBP@mSiO_2_-PEG-cRGD/I3C), an anti-tumor agent derived from food. Mediated by cRGD/integrin αvβ3 interaction, NBP@mSiO_2_-PEG-cRGD/I3C reached breast tumors in a targeted manner. Once irradiated by the near-infrared laser, our nanocarrier exhibited superior photothermal conversion, which not only induced photothermal therapy but also facilitated the release of I3C from NBP@mSiO_2_-PEG-cRGD/I3C within tumor cells to inhibit the activation of proto-oncogenic phosphoinositide 3-kinase (PI3K)-AKT signaling pathway and drive chemotherapy. All these attributes contributed to a satisfactory therapeutic effect toward breast tumors, manifesting in significant inhibition of cell proliferation, promotion of cell apoptosis, and reduction of tumor micro-vessel formation, which led to the efficient inhibition of tumor growth. Collectively, the nanocarrier developed here provided useful insights into the development of multifunctional platforms to effectively combat cancer.

## Highlights

• A multifunctional nanocarrier was rationally designed and developed.
• The nanocarrier could load indole-3-carbinol (NBP@mSiO_2_-PEG-cRGD/I3C).
• NBP@mSiO_2_-PEG-cRGD/I3C exhibited the photothermal-controlled release property.
• With laser irradiation, NBP@mSiO_2_-PEG-cRGD/I3C realized chemo-photothermal therapy.
• Under laser irradiation, NBP@mSiO_2_-PEG-cRGD/I3C suppressed 4T1 tumor growth.

## Graphical abstract

**Figure:**
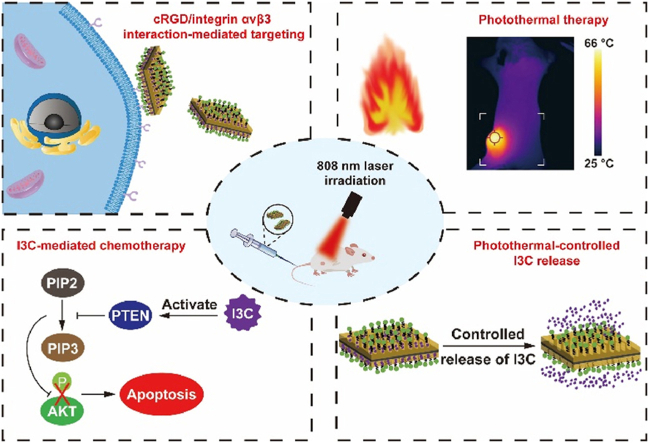

## 1 Introduction

Globally, female breast cancer accounts for 31 % of cancer incidence and 15 % of mortality, raising it a significant health concern [1]. Although current strategies, including cytoreductive surgery [2], radiotherapy [3], endocrine factor therapy [4], and chemotherapy [5], for the purpose of anti-breast cancer have demonstrated certain therapeutic effectiveness, they are always inevitably accompanied with drawbacks of serious side effects, poor controllability, and inferior efficacy. In this regard, the proposal and application of intelligent nanocarriers have been put forth as a solution to address the limitations of traditional anti-cancer solutions [[6], [7], [8], [9]]. This advancement is expected to significantly enhance the effectiveness of breast cancer treatment by delivering anti-cancer agents to tumors in a controlled and targeted way, avoiding the non-specific diffusion to maximize the presence of agents to tumors. Besides, other than just being a delivery “vehicle”, nanocarrier itself has been explored as an “agent” to impart other therapeutic modalities, realizing multimodal therapy to advance the anti-tumor effect [10]. In sum, the nanocarrier with attributes of tumor targeting, controlled release of loaded anti-tumor agents, and multimodal therapy demonstrates to be a promising strategy for effective breast cancer treatment.

Photothermal therapy (PTT) has become a prominent anti-cancer treatment due to its high therapeutic effectiveness and precise nature [11]. PTT adopts photothermal agents (PTAs) that convert light to heat when exposed to specific wavelength laser, leading to thermal ablation of tumor cells [12]. Among PTAs, black phosphorus (BP) is a kind of two-dimensional material known for its exceptional photothermal conversion efficiency [13]. Moreover, BP exhibits superior biocompatibility and can be gradually degraded into biocompatible phosphates within living organisms [14]. The aforementioned fascinating properties make it suitable to be adopted as the substrate for the construction of a nanocarrier for anti-tumor applications, which should not only integrate with the ability of PPT, but also hold the potential to release loaded agents in a controlled manner manipulated by localized heats generated by photothermal conversion [15]. However, BP is not stable in the physiological environment and is hard to modify, thus limiting its anti-tumor applications [16]. To tackle these problems, encapsulation of BP within a physiologically stable shell with numerous active modifications as well as with pore channels appears to be an effective solution. Other than inheriting all attributes of BP, such structure also imparts pore channels as the “reservoir” to host more anti-cancer agents and simplify the introduction of functional components (e.g., tumor targeting moiety), both of which would benefit the therapeutic process of BP-based nanocarriers.

Recent research indicated that certain functional components in food can regulate the immune system and interact with tumors, potentially exerting anti-tumor effects [17,18]. Especially, indole-3-carbinol (I3C) found in cruciferous vegetables has been identified as a potent anti-tumor compound that is less likely than conventional chemotherapeutic drugs to elicit adverse effects [19,20]. To be more specific, WW domain-containing E3 ubiquitin protein ligase 1 (WWP1) is genetically amplified and frequently overexpressed in cancerous tissues, resulting in pleiotropic inactivation of phosphatase and tensin homolog (PTEN). I3C has the ability to inactivate the pharmacological activity of WWP1 and reactivate PTEN, effectively inhibiting the proto-oncogenic phosphoinositide 3-kinase (PI3K)-AKT pathway that drives tumorigenesis [21]. Owing to the above mechanism, numerous cellular and animal experiments have verified that I3C can effectively attenuate or inhibit the development of diverse tumors, such as breast cancer, prostate cancer, colon cancer, lung cancer, and liver cancer [[22], [23], [24]]. Nevertheless, the inferior *in vivo* pharmacokinetics of I3C inevitably leads to non-specific diffusion within the body and clearance by reticuloendothelial systems (e.g., liver). This poses a potential risk to normal tissues and lowers the concentration of I3C at the tumor site, thereby severely comprising the therapeutic efficacy, which necessitates tumor-targeting delivery. To realize the tumor-targeting delivery, attachment of the moiety specifically binding receptors overexpressed on tumors or within the tumor microenvironment (TME) onto the nanocarrier is a straightforward and efficient way. Among these receptors, αvβ3 integrin is highly expressed in a broad spectrum of cancers but is rarely expressed on normal cells, which is associated with tumor growth, metastasis, and tumor angiogenesis [25], thus rendering it a suitable “dart” to design a nanocarrier to target [26].

**Scheme 1:**
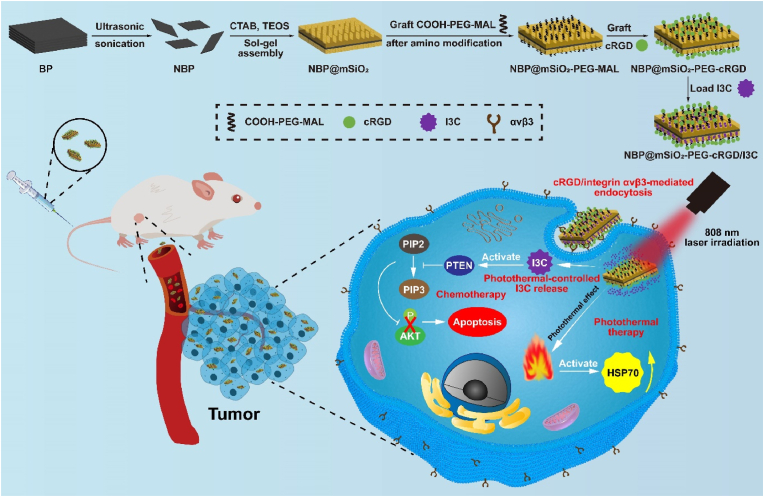
Schematic illustration of NBP@mSiO_2_-PEG-cRGD/I3C for tumor-targeting chemo-photothermal therapy in a controlled manner. NBP@mSiO_2_-PEG-cRGD was firstly synthesized by wrapping NBP within mSiO_2_ modified with cRGD, followed by loading I3C into pore channels within mSiO_2_ to obtain NBP@mSiO_2_-PEG-cRGD/I3C. When the nanocomplex was intravenously injected into 4T1 breast tumor-bearing mice, it would target the tumor due to cRGD/integrin αvβ3 interaction. Afterward, under 808 nm laser irradiation, NBP@mSiO_2_-PEG-cRGD demonstrated superior photothermal conversion to simultaneously induce PTT and promote the release of I3C from NBP@mSiO_2_-PEG-cRGD/I3C at the tumor site. The released I3C initiated the chemotherapeutic process through inhibiting the phosphorylation of AKT by upregulating the expression of PTEN to restrain the activation of PI3K-AKT signaling pathway. As a result, NBP@mSiO_2_-PEG-cRGD/I3C realized precise and efficient chemo-photothermal therapy to combat 4T1 tumors.

With all aforementioned matters in concern, in this work, a nanocarrier for targeted delivery of I3C in a controlled manner was designed for chemo-photothermal therapy of breast cancer (Scheme 1). Briefly, the nanocarrier (NBP@mSiO_2_-PEG-cRGD) was fabricated by breakdown of BP into nano-sized BP (NBP), followed by the sol-gel assembly of the mesoporous silica (mSiO_2_) shell onto NBP (NBP@mSiO_2_), after which the tumor-targeting peptide c(Arg-Gly-Asp-dPhe-Cys) (cRGD) specifically binding integrin αvβ3 expressed on breast tumor cells [27] was covalently grafted onto NBP@mSiO_2_ through a polyethylene glycol (PEG) (a well-established component beneficial for improving the pharmacokinetics of nanocarriers within the body) linker (NBP@mSiO_2_-PEG-MAL) [[28], [29], [30]]. Following administration, the I3C-loaded nanocarrier (NBP@mSiO_2_-PEG-cRGD/I3C) accumulated at the tumor site in a targeted manner, after which BP converted light energy into heats under near-infrared (NIR: a window transparent for tissues [31]) laser irradiation, to facilitate the release of I3C molecules into surrounding environment, initiating the chemotherapy. Such process up-regulated the expression of PTEN protein to inhibit AKT protein activation, thereby leading to the inactivation of the PI3K-AKT signaling pathway for tumor inhibition. Furthermore, the generated heats induced a photothermal therapeutic effect on tumor tissues to combine with I3C-based chemotherapy, advancing the anti-tumor effect to realize the efficient inhibition of breast tumors in mice. This research highlights the potential of multimodal therapeutic approaches with advances of tumor-targeting and controlled release of loaded anti-tumor agents to enhance the effectiveness and precision of cancer treatment, presenting insightful prospects into cancer therapy.

## 2 Experimental

### 2.1 Chemicals and materials

BP powder was obtained from XFNANO Technology Co., Ltd. (Nanjing, China). 1-methyl-2-pyrrolidinone (NMP), dimethyl sulfoxide (DMSO), hexadecyl trimethyl ammonium bromide (CTAB), N, N-dimethylformamide (DMF), tetraethyl orthosilicate (TEOS), and (3-aminopropyl) triethoxysilane (APTES) were obtained from Aladdin-Reagent Co., Ltd. (Shanghai, China). Carboxyl-terminated polyethylene glycol-maleimide (COOH-PEG-MAL) was obtained from Ponsure Biotech Co., Ltd. (Shanghai, China). 2-morpholinoethanesulphonic acid (MES) and 1-(3-dimethylaminopropyl)-3-ethylcarbodiimide (EDC) were purchased from Sigma-Aldrich Trading Co., Ltd. (Shanghai, China). Dulbecco's modified eagle's medium (DMEM), Roswell Park Memorial Institute (RPMI) 1640 medium, 1 % penicillin/streptomycin (v/v), fetal bovine serum (FBS), and phosphate-buffered saline (PBS) were obtained from Wuhan Servicebio Technology Co., Ltd. (Wuhan, China). cRGD was obtained from Bankpeptide Biological Technology Co., Ltd. (Hefei, China). Cell Counting Kit-8 (CCK-8) was obtained from Yeasen Biotechnology Co., Ltd. (Shanghai, China). Calcein acetoxymethyl ester (Calcein-AM)/propidium (PI) was obtained from Beyotime Biotechnology Co., Ltd. (Shanghai, China). 5, 5′, 6, 6′-tetrachloro-1, 1′, 3, 3′-tetraethyl-imideacarbocyanine iodide (JC-1) was obtained from Solarbio (Beijing, China).

### 2.2 Cell lines and animals

Human embryonic kidney 293T (HEK293T), human breast tumor cells (MCF-7), human breast tumor cells (MDA-MB-231), and mouse breast tumor cells (4T1) were purchased from Procell Life Science & Technology Co., Ltd. (Wuhan, China). Among them, except for the 4T1 cell line cultured in DMEM medium, the other three cell lines were all cultured in RPMI 1640 medium, both of which were supplemented with 1 % penicillin/streptomycin (v/v) and 10 % FBS. All cells were cultured in a humidified atmosphere containing 5 % CO_2_ at 37 °C.

BALB/c mice (female, 5 weeks, about 18 g) were obtained from Gempharmatech Co., Ltd (Jiangsu, China) and raised under standard animal raising conditions in the Laboratory Animals Department of Nanchang University (Nanchang, China). All animal studies were conducted in accordance with the regulations approved by the Animal Care and Use Committee of Nanchang University (Approval number: SYXK (Gan) 2021-0004).

### 2.3 Synthesis of NBP@mSiO_2_

50 mg of BP was dissolved in 25 mL of NMP to prepare a homogeneous BP suspension, followed by sonication at 600 W for 7 h, and then centrifugation at 5000 rpm for 5 min to obtain the NBP in the supernatant. The pH of the NBP solution was adjusted to 12 by adding 2 M NaOH, then 372 mg CTAB was added and stirred until the solution was homogenous and transparent. Subsequently, 200 μL of TEOS was added dropwise into it and stirred continuously in a mechanical stirrer (350 rpm, 45 °C) for 12 h. Finally, the product NBP@mSiO_2_ was collected by centrifugation at 10,000 rpm for 5 min, followed by washing it with a mixture of ethanol/acetic acid (8/2, v/v) for five times, and then with absolute ethanol for three times.

### 2.4 Synthesis of NBP@mSiO_2_-PEG-MAL

30 mg of NBP@mSiO_2_ was dispersed in 25 mL of isopropanol, and then 300 μL of APTES was added dropwise. The mixture was stirred for 24 h at room temperature, and then centrifuged at 10,000 rpm for 5 min to collect the precipitate. The precipitate was washed three times with ultrapure water and then three times with absolute ethanol, and finally dried in a vacuum dryer at 50 °C to obtain NBP@mSiO_2_-NH_2_. Next, 20 mg of NBP@mSiO_2_-NH_2_ was placed in a 100 mL flask, and 15 mL of MES solution (1.95 mg/mL, pH 6.5) was added, followed by sonication for 30 min. Then, 20 mg of COOH-PEG-MAL (PEG molecular weight is 2000) was dissolved in 10 mL of 0.05 M EDC (dissolved in MES) and slowly added to the above solution. The reaction mixture was sonicated for 15 min and then gently vortexed for 2 h at room temperature. Finally, the product was washed twice with ultrapure water and then with absolute ethanol before being vacuum dried at 50 °C to obtain NBP@mSiO_2_-PEG-MAL.

### 2.5 Synthesis of NBP@mSiO_2_-PEG-cRGD

cRGD (4 mM) was added to a DMF dispersion of 1 mg/mL of NBP@mSiO_2_-PEG-MAL and the mixture was processed with stirring for 12 h at room temperature. Precipitates were collected by centrifugation at 10,000 rpm and then washed three times with ultrapure water and absolute ethanol to obtain NBP@mSiO_2_-PEG-cRGD.

### 2.6 Physical characterizations of NBP@mSiO_2_-PEG-cRGD

Chemical bonds and functional groups of nanoparticles (NPs) (i.e., NBP, NBP@mSiO_2_, NBP@mSiO_2_-PEG-MAL, and NBP@mSiO_2_-PEG-cRGD) were analyzed by Fourier transform infrared spectrometer (FTIR) spectrometer (Nicolet iS50, Thermo Fisher Scientific, USA). The ultraviolet–visible–near-infrared (UV–VIS–NIR) absorption spectra of NPs (i.e., NBP and NBP@mSiO_2_-PEG-cRGD) at an equivalent concentration of NBP (100 μg/mL) were also measured by a UV spectrophotometer (Cary 60 UV–VIS, Agilent Technologies, USA). In addition, their morphology was observed by scanning electron microscope (SEM) (SU8100, Hitachi Corporation, Japan), atomic force microscopy (AFM) (Dimension Icon, Bruker Malaysia Sdn Bhd, Malaysia), and transmission electron microscope (TEM) (FEI TECNAI G2 F20, FEI Company, USA), respectively. Finally, the thermogravimetric analysis (TGA) of NBP@mSiO_2_-PEG-MAL and NBP@mSiO_2_-PEG-cRGD were performed by a thermogravimetric analyzer (TGA4000, Platinum Elmer Company, USA) to investigate the relationship between mass change and temperature.

### 2.7 Photothermal effect analysis of NBP@mSiO_2_-PEG-cRGD

Different nanomaterials (i.e., BP, NBP, NBP@mSiO_2_, NBP@mSiO_2_-PEG-MAL, and NBP@mSiO_2_-PEG-cRGD) containing the equivalent amount of BP were irradiated with an 808 nm laser at 2 W/cm^2^ for 10 min, and then the temperature change was measured by a forehead temperature gun every 100 s. In addition, the photothermal conversion of NBP@mSiO_2_-PEG-cRGD at different concentrations (i.e., 0, 50, 100, and 200 μg/mL) and NBP@mSiO_2_-PEG-cRGD (100 μg/mL) at different laser powers (i.e., 0.5, 1.0, 1.5, 2.0, 2.5, and 3.0 W/cm^2^) were also measured through the same measurement method, respectively.

To further assess its photothermal stability, PBS or NBP@mSiO_2_-PEG-cRGD (100 μg/mL) was irradiated with an 808 nm laser at 2 W/cm^2^ for 5 min, followed by cooling for 5 min, of which the whole process was performed five times. And temperature changes were recorded.

### 2.8 Determination of loading capacity of I3C by NBP@mSiO_2_-PEG-cRGD

To maximize the loading amount of I3C, different concentrations of I3C (i.e., 100, 200, 300, 400, and 500 μg/mL) were added to 100 μg/mL of NBP@mSiO_2_-PEG-cRGD and the mixture was incubated for 24 h under the dark condition. The mixture was then centrifuged at 10,000 rpm for 5 min to collect the supernatant, which was then measured at 280 nm by an ultraviolet–visible (UV–VIS) absorption spectrometer to determine the maximum loading amount of I3C by NBP@mSiO_2_-PEG-cRGD.

To determine the dynamic loading kinetics of I3C by NBP@mSiO_2_-PEG-cRGD, 200 μg/mL of I3C was added to 100 μg/mL of NBP@mSiO_2_-PEG-cRGD and incubated under dark conditions. At the determined timepoints (i.e., 0 h, 0.5 h, 1 h, 1.5 h, 2 h, 4 h, 8 h, 12 h, and 24 h), loading amount of I3C was determined according to the above description.

### 2.9 Temperature-controlled release of I3C from NBP@mSiO_2_-PEG-cRGD/I3C

1 mg of I3C dissolved in 60 μL DMSO and 500 μg of NBP@mSiO_2_-PEG-cRGD were added to a centrifuge tube containing 4 mL of PBS buffer (pH = 7.4), which was stirred for 24 h in the dark. Tubes were then placed in an ice bath for 10 min and then rinsed twice with ice PBS buffer to remove unabsorbed I3C. Finally, the precipitate of NBP@mSiO_2_-PEG-cRGD/I3C was collected by centrifugation at 10,000 rpm for 5 min. The release process was started by adding 5 mL of PBS (pH = 7.4) buffer and shaking gently at 58.7 °C and 37.0 °C, respectively. At determined timepoints, tubes were placed in cold water for 5 min, and supernatants were collected by centrifugation at 12,000 rpm. Meanwhile, based on the characteristic absorption of I3C at 280 nm, a standard curve (y = 0.0329x - 0.0101, R^2^ = 0.9998) (Fig. S1) was generated through the measurement of absorbances for different concentrations of I3C standards at this wavelength. Following this, the absorbance of the collected supernatants at 280 nm was measured, and the amounts of I3C in these supernatants were calculated according to the established I3C standard curve. Finally, the released rate of I3C was calculated according to the following formula:

$$\text{RR}\;\left(\%\right)=\frac{m_{s}}{m_{l}}\times 100\%$$

Where “RR” represents the released rate of I3C, “m_s_” and “m_l_” correspond to the amounts of I3C released into the supernatant and those loaded into NBP@mSiO_2_-PEG-cRGD, respectively.

### 2.10 Photothermal-controlled release of I3C from NBP@mSiO_2_-PEG-cRGD/I3C

For the loading process, 10 mg NBP@mSiO_2_-PEG-cRGD dispersed in 30 mL of PBS buffer (pH = 7.4) was mixed with 20 mg I3C dissolved in 1 mL of DMSO for 24 h in the dark. The solution was then washed three times with 15 mL of PBS buffer to remove unabsorbed I3C, thereby obtaining the NBP@mSiO_2_-PEG-cRGD/I3C. For photothermal-controlled release, divide the as-obtained NBP@mSiO_2_-PEG-cRGD/I3C into two groups (laser irradiation group and no laser irradiation group), and disperse them in 10 mL of PBS (pH = 7.4) buffer at 37 °C. In the laser irradiation group, on 1 h basis, the solution was irradiated with an 808 nm laser at 2 W/cm^2^ for 10 min at the beginning of each hour and ceased the irradiation in the subsequent 50 min. Corresponding solution was taken out at the end of these two periods to determine the release of I3C. The whole photothermal-controlled release process was monitored over 12 h. For the no laser irradiation group, the protocol for the determination of I3C release was the same as that adopted in the laser irradiation group. Finally, the released rate of I3C was determined using the same method as described above.

### 2.11 Endocytosis experiments

To verify the targeting of NBP@mSiO_2_-PEG-cRGD, indocyanine green (ICG)-labeled NBP@mSiO_2_-PEG-MAL/ICG-NBP@mSiO_2_-PEG-cRGD was firstly obtained through incubating NBP@mSiO_2_-PEG-MAL/NBP@mSiO_2_-PEG-cRGD with ICG-N-hydroxysuccinimide ester (ICG-NHS) in PBS at room temperature for 12 h to label ICG onto NBP@mSiO_2_-PEG-MAL/NBP@mSiO_2_-PEG-cRGD, which was performed through amidation reaction between residual -NH_2_ on NBP@mSiO_2_-PEG-MAL or NBP@mSiO_2_-PEG-cRGD and -NHS. And then 4T1 and HEK293T cells were seeded into 96-well plates containing 100 μL of medium with a density of 1 × 10^4^ cells/well for 12 h of incubation, respectively. Next, ICG-NBP@mSiO_2_-PEG-MAL and ICG-NBP@mSiO_2_-PEG-cRGD were added for another 6 h of incubation, respectively. Finally, cells were washed with PBS for three times and then imaged by a fluorescence microscopy.

### 2.12 *In vitro* biocompatibility

96-well plates with a density of 1 × 10^4^ 4T1, MDA-MB-231, MCF-7, or HEK293T cells/well were placed in a humidified environment with 5 % CO_2_ at 37 °C for 12 h. After incubating with different concentrations (i.e., 25, 50, 75, 100, 125, 150, and 200 μg/mL) of NBP@mSiO_2_-PEG-cRGD for different time, respectively, the cell viability was determined by the CCK-8 method.

### 2.13 *In vitro* cytotoxicity

A 96-well plate with a density of 1 × 10^4^ 4T1 or MDA-MB-231 cells/well was placed in a humidified environment of 5 % CO_2_ at 37 °C for 12 h. Fresh medium containing different concentrations of different materials (i.e., I3C: 1.38 μg/mL, 2.75 μg/mL, and 5.50 μg/mL; NBP@mSiO_2_-PEG-cRGD: 25 μg/mL, 50 μg/mL, and 100 μg/mL; NBP@mSiO_2_-PEG-cRGD/I3C: the amounts of NBP@mSiO_2_-PEG-cRGD and I3C in NBP@mSiO_2_-PEG-cRGD/I3C were equivalent to those in the NBP@mSiO_2_-PEG-cRGD and free I3C groups, respectively) was added for 6 h of incubation with 4T1 cells or MDA-MB-231 cells, followed by irradiating NBP@mSiO_2_-PEG-cRGD or NBP@mSiO_2_-PEG-cRGD/I3C for 10 min with the laser intensity of 2 W/cm^2^ 24 h post-treatment, the cell viability was determined by the CCK-8 method.

### 2.14 Live/dead cell staining

A 96-well plate with a density of 1 × 10^4^ 4T1 or MDA-MB-231 cells/well was placed in a humid environment with 5 % CO_2_ at 37 °C for 12 h. And then cells were treated with 100 μL fresh medium containing different formulations (i.e., PBS, I3C, NBP@mSiO_2_-PEG-cRGD/I3C, and NBP@mSiO_2_-PEG-cRGD) at an equivalent concentration of NBP (100 μg/mL) (Free I3C concentration was equivalent to the amount of I3C loaded into NBP@mSiO_2_-PEG-cRGD/I3C) for 6 h of incubation, followed by laser irradiation or not. Finally, these cells were stained with Calcein-AM/PI staining solution for 30 min, followed by two times wash with PBS and observation under a fluorescence microscope.

### 2.15 Detection of MMP changes within tumor cells

4T1 and MDA-MB-231 cells were plated into 96-well plates at a density of 1 × 10^4^ cells/well for 12 h of incubation in a humid environment at 37 °C with 5 % CO_2_, respectively. Fresh medium containing either I3C, NBP@mSiO_2_-PEG-cRGD/I3C, or NBP@mSiO_2_-PEG-cRGD was added at an equivalent concentration of NBP (100 μg/mL) (Free I3C concentration was equivalent to the amount of I3C loaded into NBP@mSiO_2_-PEG-cRGD/I3C), and after incubation for 6 h, NBP@mSiO_2_-PEG-cRGD- and NBP@mSiO_2_-PEG-cRGD/I3C-based treatments were irradiated with an 808 nm laser at 2 W/cm^2^ for 10 min, and then incubated for another 2 h. JC-1 solution was added to stain cells for 20 min at 37 °C and rinsed with PBS, and finally cells were observed by fluorescence microscopy.

### 2.16 *In vivo* biocompatibility of NBP@mSiO_2_-PEG-cRGD

100 μL of PBS and NBP@mSiO_2_-PEG-cRGD (50 mg/kg body weight) were respectively injected into mice (n = 6) via the tail vein. After 14 days, mice were dissected and major organs (i.e., heart, liver, spleen, and kidneys) were collected for calculation of organ indices, hematoxylin and eosin (H&E) staining, and measurement of serum biochemical indices, including alkaline phosphatase (ALP), alanine transaminase (ALT), aspartate aminotransferase (AST), creatine kinase (CK), and creatinine (CREA).

### 2.17 *In vivo* biodistribution of NBP@mSiO_2_-PEG-cRGD

1 mg of ICG-NHS dye was incubated with 5 mg of NBP@mSiO_2_-PEG-MAL and 5 mg of NBP@mSiO_2_-PEG-cRGD in 2 mL of PBS for 24 h at 80 rpm under dark condition, respectively. And the ungrafted ICG-NHS was removed by discarding the supernatant through multiple centrifugations until the supernatant was colorless. A subcutaneous tumor model was established by inoculating 4T1 cells (1 × 10^6^ cells/mouse) on the back of the right thigh of 5-week-old female BALB/c mice. When the tumor size of each mouse reached about 150 mm^3^ (tumor volume was calculated using the following formula: (tumor volume; mm^3^) = (tumor width; mm)^2^ × (tumor length; mm)/2 [32]), mice were injected with the corresponding material via tail vein for fluorescence imaging (n = 3). *In vivo* NIR-II fluorescence imaging was performed using a NIR-II imaging setup (NBIM-0002, Nirmidas Biotech, USA), with imaging photographs taken at predetermined timepoints.

### 2.18 *In vivo* photothermal imaging

A subcutaneous tumor model was established by inoculating 1 × 10^6^ 4T1 cells on the back of the right thigh of 5-week-old female BALB/c mice. When the tumor size of each mouse reached about 100 mm^3^, mice were injected with PBS, NBP@mSiO_2_-PEG-MAL, and NBP@mSiO_2_-PEG-cRGD via tail vein at an equivalent concentration of NBP (50 mg/kg) (n = 3), respectively. And according to *in vivo* fluorescence imaging results, after injecting materials for 24 h, tumors were irradiated with an 808 nm laser at 2 W/cm^2^ for 5 min, and photothermal imaging was performed every 30 s.

### 2.19 *In vivo* tumor therapy

4T1 cells (1 × 10^6^ cells/mouse) were injected into the left subcutaneous tissue of BALB/c mice to induce tumor growth. Once tumors reached the size of 150 mm^3^, mice were randomly assigned to 5 groups and injected with different materials (i.e., PBS, I3C, NBP@mSiO_2_-PEG-cRGD, and NBP@mSiO_2_-PEG-cRGD/I3C) via tail vein at an equivalent concentration of NBP (50 mg/kg) (Free I3C concentration was equivalent to the amount of I3C loaded into NBP@mSiO_2_-PEG-cRGD/I3C), followed by laser irradiation or not at 24 h post injection, which were denoted as the PBS group, I3C group, NBP@mSiO_2_-PEG-cRGD/I3C group, NBP@mSiO_2_-PEG-cRGD-L group, and NBP@mSiO_2_-PEG-cRGD/I3C-L group, where “L” represented laser irradiation. Tumor sizes and mouse weights were monitored for 14 days, followed by excision of tumors for histological and immunostaining analysis.

### 2.20 Sample preparation for proteomic analysis

About 200 mg fresh frozen tumor tissue were cut into pieces, washed twice with ice-cold PBS, and lysed by adding 2 mL radio immunoprecipitation assay (RIPA) lysis buffer containing 1 × protease inhibitors. Lysis was promoted by homogenization in the ice-water bath for 3 min using a handheld homogenizer (IKA T10) and sonication at 4 °C for 15 min using an ultrasonic homogenizer (SCIENTZ-IID) for 30 rounds on ice (T on = 4 s, T off = 4 s, 200 W). The lysate was centrifuged at 12,000 g and 4 °C for 10 min, the supernatant was collected and mixed with 5 vol of ice-cold acetone/ethanol/acetic acid (v/v/v = 50/50/0.1) at −20 °C overnight for protein precipitation. The precipitated protein sample was centrifuged at 20,000 g and 4 °C for 30 min, and washed with acetone and 75 % ethanol in turn. The protein sample was re-dissolved into 8 M urea/100 mM NH_4_HCO_3_ solution and the protein concentration was determined by bicin-choninic acid (BCA) assay. Then, proteins were reduced by 5 mM tris (2-carboxyethyl) phosphine (TCEP) at 25 °C for 30 min and alkylated by 10 mM iodoacetic acid (IAA) in the darkness at 25 °C for 30 min. After diluting the urea concentration to 1 M with 100 mM NH_4_HCO_3_ solution, proteins were digested with trypsin/Lys-C mix overnight at 37 °C with a 1:50 enzyme-to-protein ratio. The digestion was stopped by adding formic acid (FA) until the pH of solution was 3.0. The resulting peptides were purified by using self-made C18 cartridges, dried in a SpeedVac, and resuspended in 0.1 % FA. After estimating the concentration using a NanoDrop device (Thermo Fisher Scientific), samples were adjusted to 0.5 μg/μL with 0.1 % FA, of which 2 μL (1 μg) was injected into the mass spectrometer. Besides, for spectral library generation, a masterpool sample was prepared by combining all the peptide samples.

### 2.21 LC-MS/MS analysis and database searching

All LC-MS/MS runs were acquired using an Orbitrap Fusion Lumos Tribrid mass spectrometer (Thermo Fisher Scientific) coupled with an Easy nLC 1200 nanoflow liquid chromatography system (Thermo Fisher Scientific). Peptides were separated on a reversed-phase C18 column (20 cm × 150 μm I.D., 2.4 μm). Mobile phase A was water containing 0.1 % (v/v) FA, and mobile phase B was 80 % acetonitrile aqueous solution containing 0.1 % (v/v) FA. Peptides were separated running a gradient of 5–40 % mobile phase B over 70 min at a constant flow rate of 500 nL/min. Each sample was acquired in data-independent acquisition (DIA) mode with spray voltage of 2.3 kV and the RF lens of 30 %. The scan sequence was initiated with MS1 scans from 380 to 980 *m*/*z* recorded at 120,000 resolution, with an AGC target of 2 × 10^6^ and a maximum injection time of 50 ms. For MS2 scans, the mass range was divided into 30 segments of fixed width, and fragmentation was induced with higher energy collision dissociation (HCD) using collision energy of 30 %. Each MS2 scan was recorded at a resolution of 30,000, with an automatic gain control (AGC) target of 2.5 × 10^5^ and a maximum injection time of 54 ms. For gas-phase fractionation (GPF) measurements, the masterpool sample was repeatedly measured. Each tSIM (targeted selected ion monitoring) scan with an isolation width of 80 *m*/*z* was followed by MS2 scans with 3 *m*/*z* isolation width over 80 *m*/*z*. A total of 8 measurements were performed to cover a scan range from 380 to 980 *m*/*z*.

The obtained RAW files were firstly converted to mzML format using MSConvert in conjunction with ProteoWizard [33]. Then, the GPF-refined DIA-NN spectral library was predicted and refined using DIA-NN (1.8.1) [34]. DIA-NN was provided with a FASTA protein database of mouse as input and neural networks were used to generate spectra and retention times for the appropriate mass range of 380–980 *m*/*z*. N-terminal methionine excision was enabled. Carbamidomethylation of cysteine was activated as fixed modification. The GPF mzML files were then used with the same settings as described in the spectral library prediction step to generate a GPF-refined library for DIA analysis. Finally, the DIA mzML files were processed by DIA-NN (1.8.1) with recommended settings. Mass ranges were set appropriately for the search space (MS1: 380 *m*/*z* to 980 *m*/*z*; MS2: 145 *m*/*z* to 1450 *m*/*z*) and retention time (RT) profiling was activated. Protein and precursor false discovery rate (FDR) was set to 1 %. The protein quantification was based on the peak areas of peptides and proteins. Dysregulated proteins were identified with a paired Student's *t*-test. The *p*-values were calculated using a Benjamini-Hochberg (BH) procedure.

### 2.22 Statistical analysis

Data were represented as the mean ± standard deviation (SD) and analyzed by GraphPad Prism 9.5.0 The statistical significance analysis was performed by Student's *t*-test, one-way analysis of variance (ANOVA) and two-way ANOVA. ∗*p* < 0.05, ∗∗*p* < 0.01, ∗∗∗*p* < 0.001, and ∗∗∗∗*p* < 0.0001 were considered as different statistical significances. "ns" stood for no significant difference.

## 3 Results and discussion

### 3.1 Synthesis and characterization of NBP@mSiO_2_-PEG-cRGD

To synthesize NBP@mSiO_2_-PEG-cRGD, BP particles were initially crushed into NBP through repeated ultrasonic sonication. Subsequently, with TEOS as the silicon source and CTAB as the templating agent as well as the pore-forming agent, mSiO_2_ was formed on the surface of NBP (i.e., NBP@mSiO_2_) via a condensation process through the sol-gel assembly. Following this, amino groups (-NH_2_) were grafted onto the mSiO_2_ surface with APTES to obtain NBP@mSiO_2_-NH_2_, which were further reacted with COOH-PEG-MAL to form NBP@mSiO_2_-PEG-MAL via an amination reaction. Finally, cRGD with the sulfhydryl group was introduced through a Michael addition reaction with the maleimide group to obtain NBP@mSiO_2_-PEG-cRGD (Scheme 1), which was then systematically characterized.

**Fig. 1:**
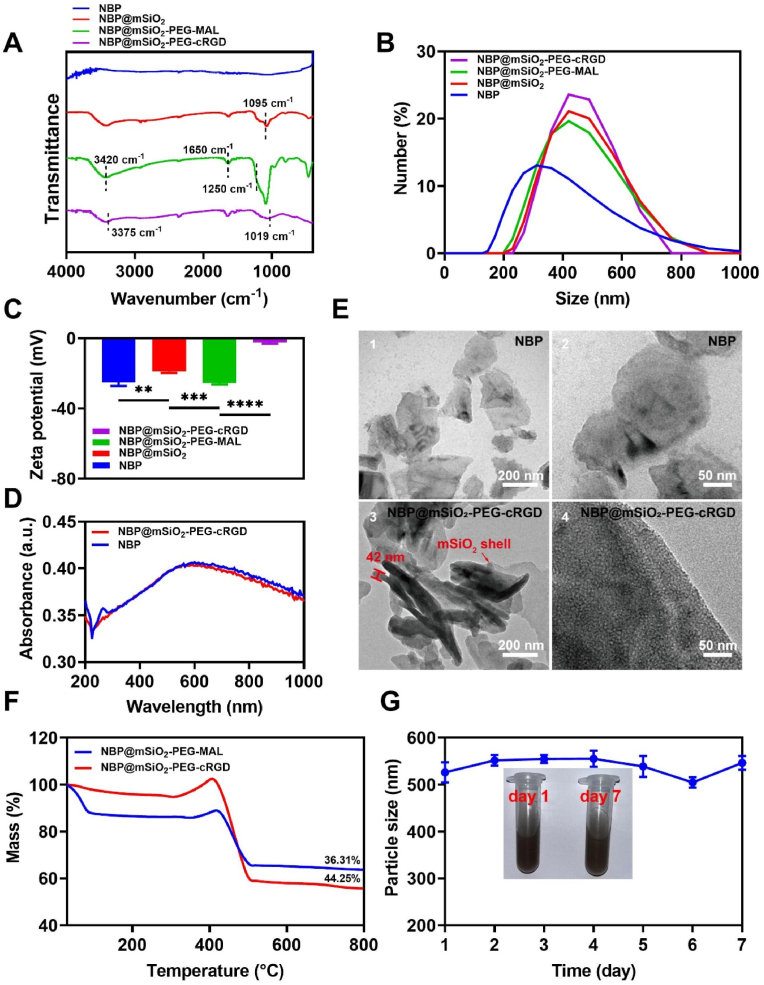
Characterizations of NBP@mSiO_2_-PEG-cRGD. (A) FTIR spectra of NBP, NBP@mSiO_2_, NBP@mSiO_2_-PEG-MAL, and NBP@mSiO_2_-PEG-cRGD. (B) Size distributions and (C) zeta potentials of NBP, NBP@mSiO_2_, NBP@mSiO_2_-PEG-MAL, and NBP@mSiO_2_-PEG-cRGD in PBS (pH = 7.4; n = 3). (D) UV–VIS–NIR spectra of NBP and NBP@mSiO_2_-PEG-cRGD. (E) TEM images of NBP (E1 and E2) and NBP@mSiO_2_-PEG-cRGD (E3 and E4). (F) TGA of NBP@mSiO_2_-PEG-MAL and NBP@mSiO_2_-PEG-cRGD. The data in the image represents the mass reduction ratios. (G) Hydrodynamic size change of NBP@mSiO_2_-PEG-cRGD in PBS (pH = 7.4) over 7 days (n = 3) and digital photos of NBP@mSiO_2_-PEG-cRGD on day 1 and day 7. Data are expressed as the mean ± SD. Statistical analysis is performed using one-way ANOVA and Student's *t*-test. ∗∗*p* < 0.01, *p* < 0.001, and ∗∗∗∗*p* < 0.0001 represent different statistical significances.

Firstly, the chemical constitution of NBP@mSiO_2_-PEG-cRGD was identified by FTIR. As shown in Fig. 1A, NBP@mSiO_2_ exhibited a strong and broad antisymmetric telescopic vibrational absorption band of Si-O-Si at 1095 cm^−1^, suggesting that NBP was encapsulated by mSiO_2_. The peaks at 1750-1650 cm^−1^ and 1350-1250 cm^−1^ represented amide I and amide II of the amide bond within NBP@SiO_2_-PEG-MAL, respectively. Besides all characteristic peaks of mSiO_2_ and the amide bond, the FTIR spectrum of NBP@mSiO_2_-PEG-cRGD showed the presence of the hydroxyl O-H stretching vibration at 3375 cm^−1^ and the carbon ring breathing vibration peak at 1019 cm^−1^ in the cyclic compound, confirming the successful grafting of cRGD. Correspondingly, X-ray photoelectron spectroscopy (XPS) showed that NBP@mSiO_2_-PEG-cRGD contained O, N, C, S, P, and Si elements (Fig. S2). With the stepwise introduction of new components, the hydration sizes of NBP (286.55 nm), NBP@mSiO_2_ (472.97 nm), NBP@mSiO_2_-PEG-MAL (492.53 nm), and NBP@mSiO_2_-PEG-cRGD (511.90 nm) increased in sequence (Fig. 1B). Meanwhile, changes in zeta potentials further evidenced the successful stepwise introduction of new components as reasonable changes in zeta potentials occurred for different materials (i.e., NBP (−25.10 mV), NBP@mSiO_2_ (−18.95 mV), NBP@mSiO_2_-PEG-MAL (−25.46 mV), and NBP@mSiO_2_-PEG-cRGD (−2.23 mV)) due to the positively charged attribute of -NH_2_ and the negatively charged attribute of cRGD (Fig. 1C). The UV–VIS–NIR spectrum of NBP@mSiO_2_-PEG-cRGD showed absorption characteristics similar to NBP within the NIR region (780 nm–900 nm) (Fig. 1D), indicating the photothermal conversion capability of NBP@mSiO_2_-PEG-cRGD under NIR laser irradiation (a transparent window for tissue). As intuitively revealed by SEM (Figs. S3, S4, and S5), AFM (Fig. S6), and TEM (Fig. 1E) analysis, NBP displayed a sheet-like structure with size of approximately 300 nm and a thickness of approximately 1.7 nm, and NBP@mSiO_2_-PEG-cRGD possessed a porous layered structure with the thickness of about 42 nm, directly confirming the formation of SiO_2_ shell. Furthermore, a comparison with NBP@mSiO_2_-PEG-MAL revealed that the weight ratio of cRGD grafted onto NBP@mSiO_2_-PEG-cRGD was about 10 %, securing the premise for tumor targeting (Fig. 1F). In addition, the negligible change of size (Fig. 1G) and zeta potential (Fig. S7) of NBP@mSiO_2_-PEG-cRGD over 7 days in PBS (pH = 7.4) indicated the stability of NBP@mSiO_2_-PEG-cRGD in the physiological environment, possibly due to the introduction of PEG [35]. Collectively, all above characterizations demonstrated the successful fabrication of NBP@mSiO_2_-PEG-cRGD, providing a solid basis for subsequent *in vitro* and *in vivo* studies.

### 3.2 Evaluation of functionalities of NBP@mSiO_2_-PEG-cRGD

**Fig. 2:**
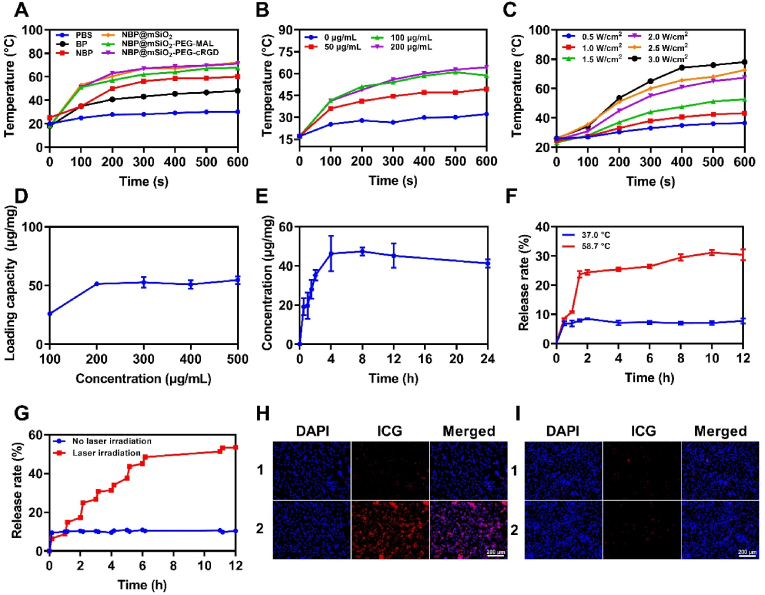
Functionalities of NBP@mSiO_2_-PEG-cRGD. (A) Temperature change over time in different material solutions at an equivalent concentration of BP under 2 W/cm^2^ 808 nm laser irradiation. (B) Photothermal response of NBP@mSiO_2_-PEG-cRGD with different concentrations (i.e., 0, 50, 100, and 200 μg/mL) under 808 nm laser irradiation at 2 W/cm^2^. (C) Photothermal response of NBP@mSiO_2_-PEG-cRGD at a concentration of 100 μg/mL under 808 nm laser irradiation with different laser power densities (i.e., 0.5, 1.0, 1.5, 2.0, 2.5, and 3.0 W/cm^2^). (D) Evaluation of the loading capacity of I3C by NBP@mSiO_2_-PEG-cRGD. (E) Dynamic loading of I3C by NBP@mSiO_2_-PEG-cRGD. (F) The cumulative release curves of I3C at different temperatures (i.e., 37.0 °C and 58.7 °C). (G) The release of I3C from NBP@mSiO_2_-PEG-cRGD/I3C manipulated by 808 nm laser irradiation or not. Fluorescence microscope images of (H) 4T1 cells and (I) HEK293T cells after co-incubation with ICG-NBP@mSiO_2_-PEG-MAL (1) and ICG-NBP@mSiO_2_-PEG-cRGD (2) for 6 h, respectively; Blue: DAPI; Red: ICG; Scale bar = 200 μm. (For interpretation of the references to color in this figure legend, the reader is referred to the Web version of this article.)

NBP@mSiO_2_-PEG-cRGD designed for anti-breast cancer theoretically possessed advances of photothermal effects, high-efficiency loading of anti-tumor agents, controlled release of loaded agents, and tumor cell targeting, which were then evaluated in following studies. Firstly, the photothermal conversion capability of NBP@mSiO_2_-PEG-cRGD, the prerequisite for inducing PTT, was assessed. As shown in Fig. 2A and S8, with the prolonging of irradiation time using an 808 nm laser (a wavelength transparent for tissues) [31], the temperature of PBS solution slightly increased, while those of PBS solutions containing BP, NBP, NBP@mSiO_2_, NBP@mSiO_2_-PEG-MAL, or NBP@mSiO_2_-PEG-cRGD (all materials contained the equivalent amount of BP) increased in a rapid manner. Specifically, the nano-miniaturization of BP would enhance its photothermal conversion ability as revealed by the higher temperature increase rates of NBP-based materials (i.e., NBP, NBP@mSiO_2_, NBP@mSiO_2_-PEG-MAL, or NBP@mSiO_2_-PEG-cRGD), possibly due to the enhanced light absorption and resonance absorption [36]. Moreover, the temperature of NBP@mSiO_2_-PEG-cRGD suspension rose to 71.2 °C after 600 s of laser irradiation, far exceeding the cell death threshold of 43 °C [37]. In addition, following a one-week storage in PBS, the photothermal effects of NBP and NBP@mSiO_2_ were re-evaluated. It was found that the photothermal effects of NBP and NBP@mSiO_2_ were comparable to those depicted in Fig. 2A and S8, featuring similar temperature-raising curves (Fig. S9). This finding indicated that NBP-based materials possessed favorable stability, a key requirement for effective photothermal therapy. Subsequently, the photothermal conversion capability of NBP@mSiO_2_-PEG-cRGD was systematically evaluated at different concentrations and laser power densities. The results showed that as the concentration of NBP@mSiO_2_-PEG-cRGD increased, the photothermal effect became more obvious, reaching a saturation when the concentration was 100 μg/mL (Fig. 2B and S10), which was possibly because the solution with material concentration beyond 200 μg/mL was too dark for the laser to completely penetrate. Afterward, we fixed the concentration of NBP@mSiO_2_-PEG-cRGD at 100 μg/mL and compared the photothermal effects of NBP@mSiO_2_-PEG-cRGD initiated by different laser powers. As disclosed in Fig. 2C, NBP@mSiO_2_-PEG-cRGD exhibited a significant temperature increase from 36.4 °C to 78.0 °C after 600 s irradiation by changing the laser power density from 0.5 W/cm^2^ to 3.0 W/cm^2^. Although 2.5 W/cm^2^ and 3.0 W/cm^2^ laser power densities contributed to excellent photothermal effects, these two laser intensities exhibited obvious harmful effects on normal tissues or cells [38]. Therefore, we chose the 2.0 W/cm^2^ laser power density for subsequent biological experiments, which had also been adopted in other research [[39], [40], [41]]. Eventually, the photothermal stability of NBP@mSiO_2_-PEG-cRGD was assessed by the irradiation and cooling cycle (irradiation for 5 min, followed by a 5-min cooling period). After 5 cycles, the photothermal conversion capability of NBP@mSiO_2_-PEG-cRGD negligibly changed (Fig. S11). Collectively, the significant photothermal conversion efficiency and superior photothermal stability indicated that the NBP@mSiO_2_-PEG-cRGD would be an excellent agent for PTT of cancer.

NBP@mSiO_2_-PEG-cRGD was expected to load I3C within pore channels through non-covalent forces (i.e., electrostatic interaction, hydrogen bonding, and Van der Waals force) and control its release in a photothermally responsive manner due to the heat-induced dissociation of non-covalent forces, thereby exerting chemotherapeutic effects on tumors [42]. To evaluate the capacity of NBP@mSiO_2_-PEG-cRGD to load I3C, I3C with various concentrations (i.e., 100, 200, 300, 400, and 500 μg/mL) was co-incubated with NBP@mSiO_2_-PEG-cRGD at the concentration of 100 μg/mL. The results showed that the addition of 200 μg/mL of I3C saturated the NBP@mSiO_2_-PEG-cRGD loading capacity, calculated to be a maximum loading amount of 55 μg/mg (Fig. 2D). In addition, the specific loading time for reaching saturation demonstrated that the loading amount of I3C stabilized and peaked after 4 h incubation (Fig. 2E). Therefore, to fabricate the NBP@mSiO_2_-PEG-cRGD/I3C with the largest loading amount of I3C, the mass ratio of NBP@mSiO_2_-PEG-cRGD to I3C and the incubation time was fixed at 1:2 and 4 h, respectively. Then, the release behavior of I3C from NBP@mSiO_2_-PEG-cRGD/I3C was firstly characterized in PBS at 37.0 °C (the temperature of normal physiological

conditions) and 58.7 °C (the temperature at which NBP@mSiO_2_-PEG-cRGD (100 μg/mL) could reach under laser irradiation as characterized above (Fig. 2B)) to explore the potential in NIR laser irradiation-induced release of loaded I3C. As shown in Fig. 2F, interestingly, a notable disparity in I3C release was observed between environmental temperatures of 37.0 °C and 58.7 °C, and the cumulative release of I3C at 58.7 °C was approximately 4 times of that at 37.0 °C. Such phenomenon indicated the controllable release of I3C by NBP@mSiO_2_-PEG-cRGD in response to different temperatures, highlighting the potential of inhibition of premature release of loaded I3C before reaching the tumor without external stimulus. Encouraged by above results, we moved forward to captivate on applying NIR laser to control the release of loaded I3C in light of the favorable photothermal effect of NBP@mSiO_2_-PEG-cRGD (Fig. 2G). To profile the release behavior, we monitored the cumulative released amounts of I3C from NBP@mSiO_2_-PEG-cRGD/I3C under initial 10 min continuous laser irradiation with subsequent 50 min’ cessation of laser irradiation on 1 h basis over 12 h. Excitingly, even with 4 times shorter release time, the cumulative release amounts of I3C under 10 min laser irradiation were significantly higher than those released during the 50 min period without laser irradiation, leading to a step-by-step release pattern. This finding emphasized the feasibility to manipulate the release of loaded I3C in a controllable manner with NIR laser stimulus, expecting to maximize the presence of I3C to the tumor and avoid the non-specific diffusion of I3C to normal tissues, both of which would advance the effectiveness of cancer therapy.

In addition, the cRGD peptide anchored on the NBP@mSiO_2_-PEG-cRGD could specifically bind integrin αvβ3 overexpressed on tumor cell surfaces, thereby enhancing the uptake of NBP@mSiO_2_-PEG-cRGD by tumor cells to advance the anti-tumor effect. To evaluate this, ICG-labeled NBP@mSiO_2_-PEG-MAL (ICG-NBP@mSiO_2_-PEG-MAL) and ICG-labeled NBP@mSiO_2_-PEG-cRGD (ICG-NBP@mSiO_2_-PEG-cRGD) (Please see Experimental Section for detailed labeling process of ICG) were co-incubated with 4T1 mouse breast tumor cells overexpressing integrin αvβ3 [27] and HEK293T cells barely expressing integrin αvβ3 [43], respectively, followed by fluorescence imaging. As verified in Fig. 2H, I, and S12, a significantly stronger red fluorescence was revealed in the cytoplasm of 4T1 cells treated with ICG-NBP@mSiO_2_-PEG-cRGD compared to those treated with ICG-NBP@mSiO_2_-PEG-MAL. In contrast, HEK293T cells exhibited very weak fluorescence after incubation of ICG-NBP@mSiO_2_-PEG-MAL or ICG-NBP@mSiO_2_-PEG-cRGD, with no significant difference between them. In conclusion, above validated multifunctionalities of NBP@mSiO_2_-PEG-cRGD would jointly benefit the therapeutic effectiveness toward breast cancer.

### 3.3 NBP@mSiO_2_-PEG-cRGD/I3C-based treatment for combating breast tumor cells *in vitro*

**Fig. 3:**
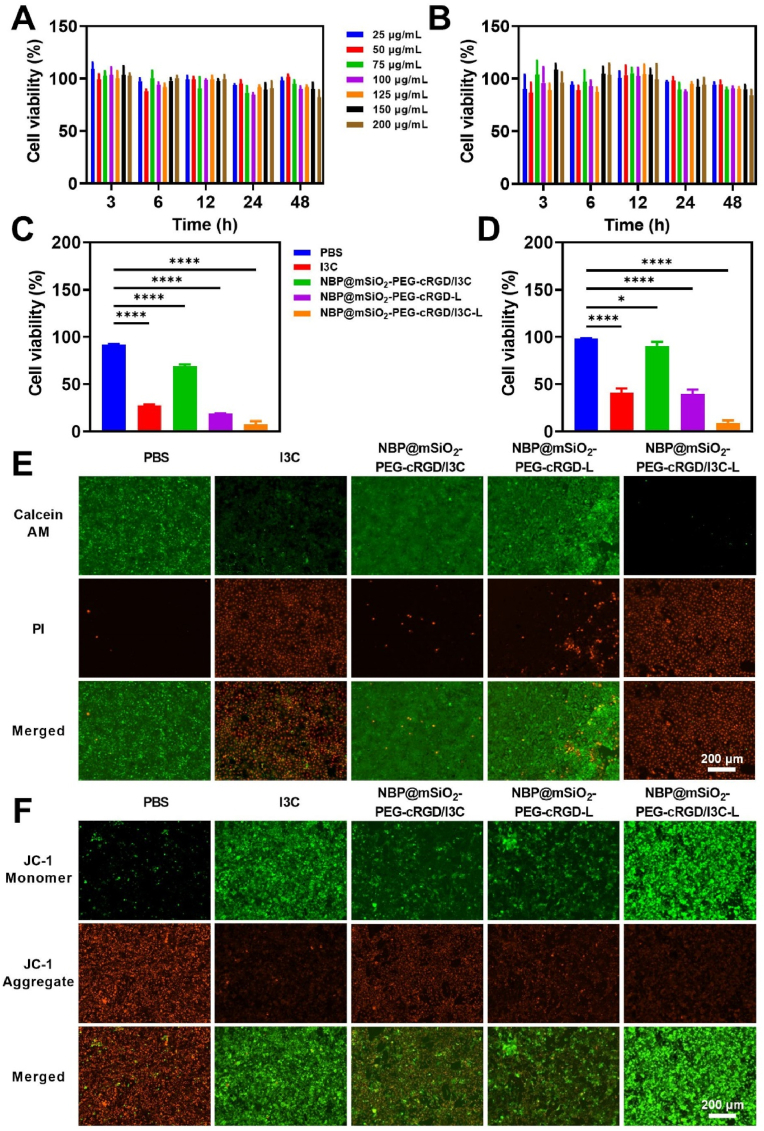
Evaluation of NBP@mSiO_2_-PEG-cRGD/I3C-based treatment against breast tumor cells. Viabilities of (A) 4T1 cells and (B) MDA-MB-231 cells treated with different concentrations of NBP@mSiO_2_-PEG-cRGD for 3 h, 6 h, and 12 h, respectively (n = 3). Viabilities of (C) 4T1 cells and (D) MDA-MB-231 cells under different treatment scenarios (i.e., PBS, I3C (5.5 μg/mL), NBP@mSiO_2_-PEG-cRGD/I3C (100 μg/mL), NBP@mSiO_2_-PEG-cRGD-L (NBP@mSiO_2_-PEG-cRGD: 100 μg/mL), and NBP@mSiO_2_-PEG-cRGD/I3C-L (NBP@mSiO_2_-PEG-cRGD/I3C: 100 μg/mL); “L” represents “808 nm laser irradiation at 2 W/cm^2^ for 10 min”). (E) Live/dead staining of 4T1 cells treated with different scenarios (i.e., PBS, I3C, NBP@mSiO_2_-PEG-cRGD/I3C, NBP@mSiO_2_-PEG-cRGD-L, and NBP@mSiO_2_-PEG-cRGD/I3C-L; Green: living cells were stained with Calcein-AM; Red: dead cells were stained with PI); Scale bar = 200 μm. (F) MMP variation characterized by JC-1 staining after treating 4T1 cells with different scenarios (i.e., PBS, I3C, NBP@mSiO_2_-PEG-cRGD/I3C, NBP@mSiO_2_-PEG-cRGD-L, and NBP@mSiO_2_-PEG-cRGD/I3C-L; Red: JC-1 aggregates, Green: JC-1 monomer); Scale bar = 200 μm. Data are expressed as the mean ± SD. Statistical analysis is performed using one-way ANOVA and Student's *t*-test. ∗*p* < 0.05 and ∗∗∗∗*p* < 0.0001 represent different statistical significances. (For interpretation of the references to color in this figure legend, the reader is referred to the Web version of this article.)

Motivated by above-verified superiorities, cytological experiments were conducted to confirm the therapeutic impact of NBP@mSiO_2_-PEG-cRGD/I3C on tumor cells. Satisfactory biocompatibility is a prerequisite for the application of nanocarriers in cancer treatment. As shown in Fig. 3A and B, NBP@mSiO_2_-PEG-cRGD showed negligible cytotoxicity to 4T1 cells, MDA-MB-231 cells, MCF-7 cells (Fig. S13), or HEK293T cells (Fig. S14) even at the high concentration of 200 μg/mL.

Subsequently, we exposed 4T1 or MDA-MB-231 breast cancer cells to various concentrations of I3C, NBP@mSiO_2_-PEG-cRGD, and NBP@mSiO_2_-PEG-cRGD/I3C to systematically assess the impact of different treatment scenarios on cell viabilities (denoted as PBS group, I3C group, NBP@mSiO_2_-PEG-cRGD/I3C group, NBP@mSiO_2_-PEG-cRGD-L group, and NBP@mSiO_2_-PEG-cRGD/I3C-L group, with “L” representing “laser irradiation”). In the case of the free anti-tumor drug I3C, an increase in concentration led to more significant toxicity, which was evident in the decreasing survival rate of 4T1 cells, confirming the I3C-mediated chemotherapy (Fig. 3C–S15, and S16). Interestingly, following the encapsulation of I3C within the pore channels of the biocompatible NBP@mSiO_2_-PEG-cRGD carrier, the resulting NBP@mSiO_2_-PEG-cRGD/I3C exhibited slight impact on cell viability within the applied concentrations (Fig. 3C–S15, and S16), which should be due to small amounts of I3C released at 37.0 °C (the cell culturing temperature). In contrast, under the action of laser irradiation, NBP@mSiO_2_-PEG-cRGD treatment demonstrated cytotoxicity that became more significant as the concentration of NBP@mSiO_2_-PEG-cRGD (Fig. 3C–S15, and S16) increased, thus corroborating the presence of PTT. Combining the chemotherapy and PTT, the lowest cell survival rate of 7.42 % was revealed in the NBP@mSiO_2_-PEG-cRGD/I3C (100 μg/mL) treatment under laser irradiation (Fig. 3C), echoing the laser irradiation-controlled release of loaded I3C. These treatment scenarios demonstrated similar trends regarding the therapeutic effects on MDA-MB-231 cells (Fig. 3D–S17, and S18), with the lowest cell viabil ity down to only 9.16 % occurring in the NBP@mSiO_2_-PEG-cRGD/I3C (100 μg/mL) treatment under laser irradiation (Fig. 3D), further highlighting the rationality of constructing NBP@mSiO_2_-PEG-cRGD/I3C for combating breast cancer under laser irradiation.

To further intuitively confirm the killing effect of NBP@mSiO_2_-PEG-cRGD/I3C on 4T1 and MDA-MB-231 tumor cells when exposed to 808 nm laser irradiation, we utilized calcein-acetoxymethyl ester (AM) (green) and propidium iodide (PI) (red) dyes to stain live and dead cells after different treatments, respectively. The results in Fig. 3E and S19 demonstrated that PBS and NBP@mSiO_2_-PEG-cRGD/I3C could only cause partial cell death. In contrast, more dead cells appeared in the NBP@mSiO_2_-PEG-cRGD-L group, and cells in the NBP@mSiO_2_-PEG-cRGD/I3C-L group almost completely died, which was consistent with the outcomes of cell viability experiments described above (Fig. 3C). To dig deeper, we detected changes in mitochondrial membrane potential (MMP), a common phenomenon happening during the apoptosis of tumor cells, which could be specifically profiled by JC-1 fluorescent probe, whose transition from aggregates (red fluorescence) to monomers (green fluorescence) represented a decrease in MMP [44]. As shown in Fig. 3F and S21, the PBS and NBP@mSiO_2_-PEG-

cRGD/I3C groups exhibited identical intensity ratio of red fluorescence to green fluorescence, while the green fluorescence became more significant in the I3C and NBP@mSiO_2_-PEG-cRGD-L groups, which was mainly caused by I3C-mediated cell apoptosis and NBP@mSiO_2_-PEG-cRGD-induced PTT, respectively. Obviously, under the combined effect, the NBP@mSiO_2_-PEG-cRGD/I3C-L group revealed the strongest green fluorescence, suggesting that most 4T1 cells were in an apoptotic state. Above results were also observed in the treatments of MDA-MB-231 cells (Figs. S20 and S22). Overall, these findings indicated that NBP@mSiO_2_-PEG-cRGD/I3C could significantly inhibit cell proliferation and promote cell apoptosis to realize efficient combat of breast tumor cells under laser irradiation.

### 3.4 *In vivo* evaluation of biocompatibility and photothermal effect of NBP@mSiO_2_-PEG-cRGD

**Fig. 4:**
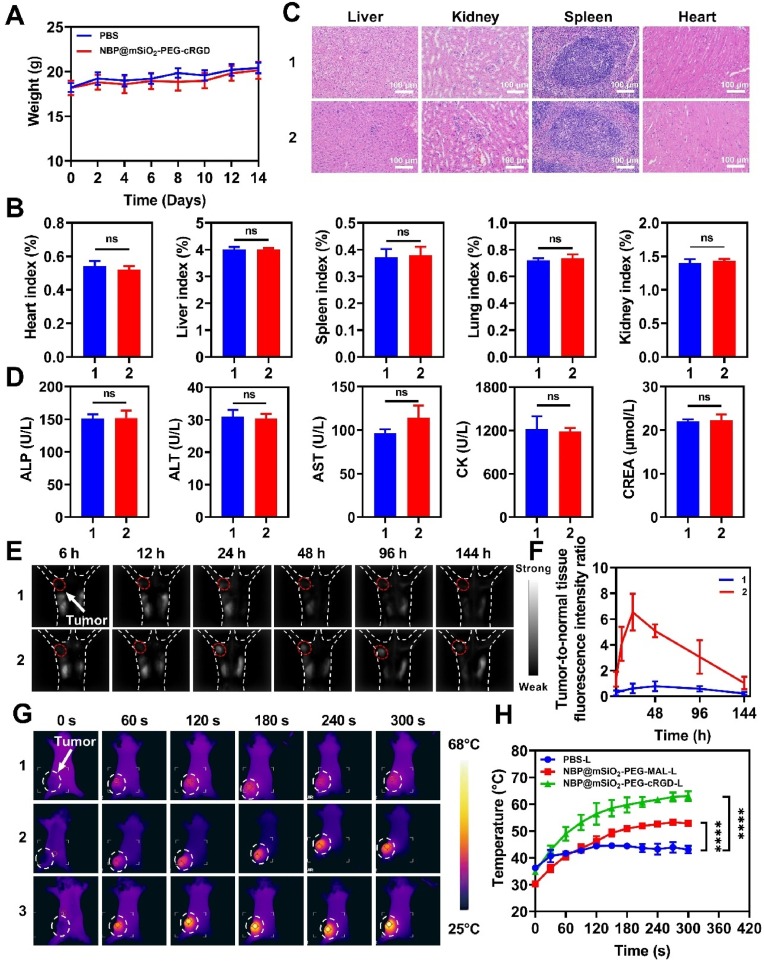
*In vivo* superior biocompatibility of NBP@mSiO_2_-PEG-cRGD and its photothermal effects within tumors. (A) Body weight change and (B) organ indices (i.e., heart index, liver index, spleen index, lung index, and kidney index) of mice with or without administration of NBP@mSiO_2_-PEG-cRGD (1: PBS, 2: NBP@mSiO_2_-PEG-cRGD). (C) H&E staining of the liver, kidney, spleen, and heart from mice with or without administration of NBP@mSiO_2_-PEG-cRGD (1: PBS, 2: NBP@mSiO_2_-PEG-cRGD); Scale bar = 100 μm (D) Serum biochemical indices (i.e., ALP, ALT, AST, CK, and CREA). of mice with or without administration of NBP@mSiO_2_-PEG-cRGD (1: PBS, 2: NBP@mSiO_2_-PEG-cRGD). (E) *In vivo* fluorescence imaging of 4T1 tumor-bearing mice after intravenous injection of ICG-NBP@mSiO_2_-PEG-MAL (1) or ICG-NBP@mSiO_2_-PEG-cRGD (2); The red circle highlights the tumor. (F) The quantitative analysis of fluorescence intensity ratio of tumors to normal tissues based on (E). (G) Temperature change of tumors in mice under various treatments as a function of irradiation time (1: PBS-L, 2: NBP@mSiO_2_-PEG-MAL-L, 3: NBP@mSiO_2_-PEG-cRGD-L; “L” represents “laser irradiation”); The white circle highlights the tumor. (H) The quantitative analysis of temperature variation of tumors based on (F). Data are expressed as the mean ± SD. Statistical analysis is performed using one-way ANOVA and Student's *t*-test. ∗∗∗∗*p* < 0.0001 represents a statistical significance; “ns” represents no significant difference. (For interpretation of the references to color in this figure legend, the reader is referred to the Web version of this article.)

The above *in vitro* superior anti-tumor effects of NBP@mSiO_2_-PEG-cRGD prompted us to proceed *in vivo* study, of which biosafety assessment was firstly evaluated. The results revealed that the body weight of mice with or without administration of NBP@mSiO_2_-PEG-cRGD remained stable for a 14-day treatment (Fig. 4A). After 14 days, all the mice were sacrificed, and their organs and blood were collected for further analysis. There were no significant differences in major organ (i.e., heart, liver, spleen, lung, and kidney) indices among all the mice (Fig. 4B). As H&E staining of these organ sections showed, no evident histopathological damages were found after NBP@mSiO_2_-PEG-cRGD treatment, manifested by clear hepatocyte structures, intact cell morphology, normal glomerular size and shape, renal interstitium without inflammatory cell infiltration, obvious central arteries and lymphocytes in the spleen section, and parallel arrangement of myocardial cells (Fig. 4C). In addition, serum biochemical markers were also measured to further determine the biological safety. The levels of ALP/ALT/AST reflecting liver function, CK reflecting myocardial injury, and CREA reflecting renal filtration function revealed no significant differences among mice treated with or without NBP@mSiO_2_-PEG-cRGD (Fig. 4D). Moreover, NBP@mSiO_2_-PEG-cRGD (as high as 300 μg/mL)-treated erythrocytes demonstrated a low rate of hemolysis (Fig. S23). Collectively, all the results suggested the superior biosafety of NBP@mSiO_2_-PEG-cRGD, suitable for subsequent *in vivo* applications.

As verified *in vitro*, the satisfactory photothermal effect of NBP@mSiO_2_-PEG-cRGD is the premise to trigger the chemo-photothermal combinatory therapy. Considering this, we began to investigate the photothermal effect of NBP@mSiO_2_-PEG-cRGD *in vivo*. Firstly, the pharmacokinetic and distribution of NBP@mSiO_2_-PEG-cRGD within the mouse body was probed in order to secure the timepoint when NBP@mSiO_2_-PEG-cRGD accumulated most within the tumor, which can guide the laser irradiation to achieve the best photothermal effect. It has been referred that taking advantage of the emission tailing into the second near-infrared (NIR-II) window (1000–1700 nm; Fig. S24), ICG can be used as a dye for high-quality *in vivo* NIR-II fluorescence imaging due to the low photon scattering and negligible autofluorescence of tissues [45,46]. For this, ICG-NBP@mSiO_2_-PEG-cRGD was intravenously injected into 4T1 tumor-bearing mice for *in vivo* NIR-II fluorescence imaging, which included ICG-NBP@mSiO_2_-PEG-MAL as a comparison. As expected, compared with ICG-NBP@mSiO_2_-PEG-MAL, tumors within mice administrated with ICG-NBP@mSiO_2_-PEG-cRGD were brighter, with peak brightness observed at 24 h post administration (Fig. 4E and F), which should be attributed to the specific recognition of integrin αvβ3 overexpressed on 4T1 breast tumors by cRGD (Fig. S25), consistent with *in vitro* results (Fig. 2H and I). Similarly, we also demonstrated this conclusion through *in vivo* fluorescence imaging based on another

commonly used fluorophore Cyanine 5 (Cy5) (Fig. S26) and cytotoxicity assays (Fig. S27), consolidating the cRGD-mediated targeting behavior.

Guided by above imaging results, we conducted *in vivo* photothermal imaging of NBP@mSiO_2_-PEG-cRGD in mice by irradiating tumors with an 808 nm laser at 24 h post injection of NBP@mSiO_2_-PEG-MAL or NBP@mSiO_2_-PEG-cRGD. As depicted in Fig. 4G and H, the temperature at tumor sites of mice treated with NBP@mSiO_2_-PEG-cRGD could reach a maximum of 63.1 °C, far exceeding the temperature threshold of 43 °C at which tumor cells began to die [47], while those administrated with PBS and NBP@mSiO_2_-PEG-MAL only rose to 43.1 °C and 52.9 °C, respectively, confirming the favorable photothermal effect of NBP@mSiO_2_-PEG-cRGD within the tumor *in vivo*. These findings co-suggested that NBP@mSiO_2_-PEG-cRGD could target tumor cells *in vivo* to promote the photothermal effect within the tumors under laser irradiation, providing a sound foundation for following controlled chemo-photothermal therapy of breast cancer.

### 3.5 *In vivo* anti-tumor effect of NBP@mSiO_2_-PEG-cRGD/I3C-based treatment

**Fig. 5:**
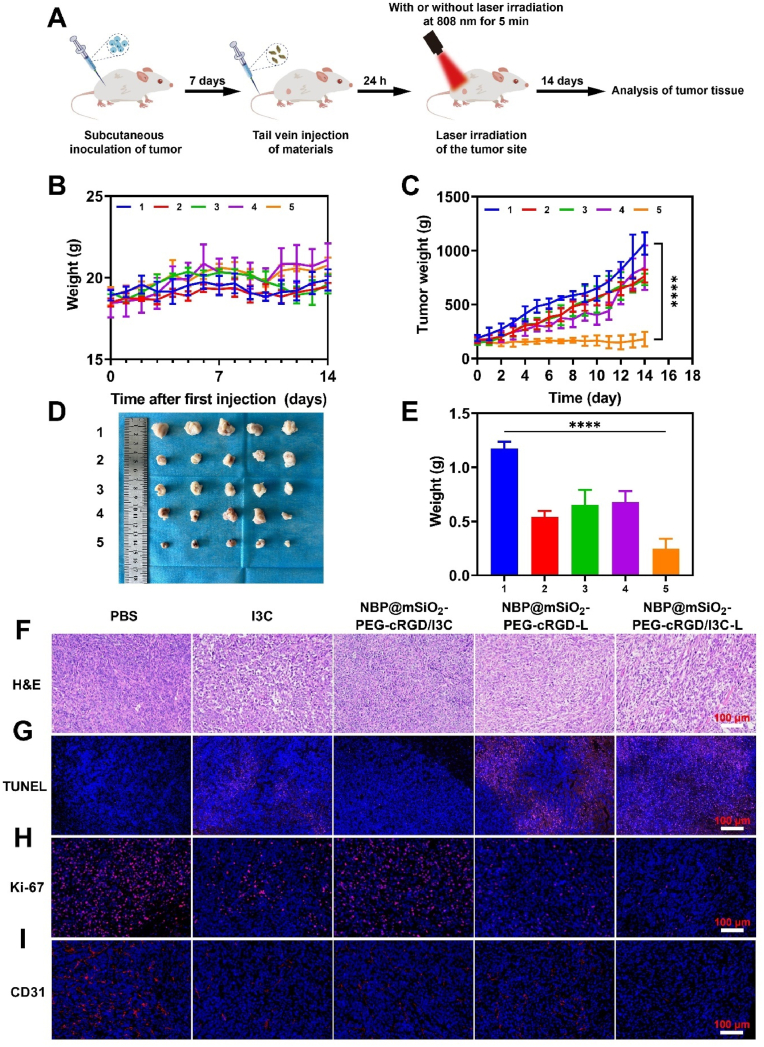
*In vivo* tumor therapy based on NBP@mSiO_2_-PEG-cRGD/I3C. (A) Schematic illustration of the treatment process. The tumor-bearing mice were established by subcutaneous injection of 4T1 cells in the right flank of mice. Seven days later, different materials were administered via tail vein injection, followed by irradiation of the tumor site with an 808 nm laser for 5 min (2 W/cm^2^) at 24 h post injection or not. The growth status of mice was monitored daily, and they were sacrificed and tumors were collected for analysis on day 14 after injection. Changes in (B) mouse body weight and (C) tumor volume in different treatment groups over the course of the treatment period. (D) Photographs and (E) weights of excised tumors from different treatment groups at 14 days post injection of different materials. (F) H&E staining, (G) TUNEL staining (Blue: DAPI for nucleus; Red: tetramethylrhodamine (TMR) for apoptotic tumor cells), (H) Ki-67 immunofluorescence staining (Blue: DAPI for nucleus; Red: Ki-67), and (I) CD31 immunofluorescence staining (Blue: DAPI for nucleus; Red: CD31) of tumor tissues from different treatment groups at the end of treatments (Scale bar = 100 μm). 1: PBS; 2: I3C; 3: NBP@mSiO_2_-PEG-cRGD/I3C; 4: NBP@mSiO_2_-PEG-cRGD-L; 5: NBP@mSiO_2_-PEG-cRGD/I3C-L; “L” represents laser irradiation. Data are expressed as the mean ± SD. Statistical analysis is performed using one-way ANOVA and Student's *t*-test. ∗∗∗∗*p* < 0.0001 represents a statistical significance. (For interpretation of the references to color in this figure legend, the reader is referred to the Web version of this article.)

Eventually, we began to evaluate the therapeutic effect of NBP@mSiO_2_-PEG-cRGD/I3C on breast tumors under laser irradiation *in vivo*, for which a 4T1 subcutaneous tumor model was established. As shown in Fig. 5A, when tumor volumes reached approximately 150 mm^3^, mice randomly divided into 5 groups were treated with different scenarios: PBS, I3C, NBP@mSiO_2_-PEG-cRGD/I3C, NBP@mSiO_2_-PEG-cRGD-L, and NBP@mSiO_2_-PEG-cRGD/I3C-L, respectively (“L” represents “laser irradiation”). According to the fluorescence imaging (Fig. 4E) performed as above mentioned, tumors were irradiated with 808 nm laser irradiation of 2 W/cm^2^ for 5 min at 24 h post administration of different formulations. As shown in Fig. 5B and S28, mouse weights and major organ indices in all five groups remained relatively stable over the treatment course of 14 days, further confirming minimal *in vivo* toxicity of these formulations. Furthermore, the change of tumor volume over time (Fig. 5C) demonstrated that compared with the PBS group, I3C or NBP@mSiO_2_-PEG-cRGD/I3C showed a finite inhibitory effect on tumor growth due to the limited amount of I3C exerting anti-tumor effects, which was caused by the poor pharmacokinetics of free I3C and insufficient release of loaded I3C within the tumor at physiological temperature (37.0 °C) as characterized above (Fig. 2F), respectively [48,49]. Under the tumor-targeting assistance of PTT, the NBP@mSiO_2_-PEG-cRGD-L group showed decent therapeutic effects in the first place (before day 11) but experienced a recovery of aggressive tumor growth in the later time, indicating sole PTT for 5 min was not enough to exert sustainable therapeutic efficacy. As expected, the most significant inhibition of tumor growth was observed in the NBP@mSiO_2_-PEG-cRGD/I3C-L group owing to the advances of targeting ability, PTT, and photothermal conversion-promoted release of I3C within the tumors for chemotherapy. This led to the smallest tumor volume of 179.3 mm^3^ and lowest tumor weight of 0.246 g at the end of treatment, in striking contrast to those observed in PBS, I3C, NBP@mSiO_2_-PEG-cRGD/I3C, and NBP@mSiO_2_-PEG-cRGD-L groups, which reached 1065.0 mm^3^/1.175 g, 763.3 mm^3^/0.539 g, 733.1 mm^3^/0.652 g, and 841.7 mm^3^/0.676 g, respectively (Fig. 5D and E).

To further confirm the superiority of NBP@mSiO_2_-PEG-cRGD/I3C-L in combating the breast tumor, more characterizations toward tumors tissues collected at the end of treatments were performed. As shown in Fig. 5F, the H&E staining results revealed that the PBS group demonstrated small intercellular spaces and high cell density, indicative of the existence of numerous tumor cells. In contrast, the I3C group, NBP@mSiO_2_-PEG-cRGD/I3C group, and NBP@mSiO_2_-PEG-cRGD-L group exhibited many vacuoles in tumor tissues, which may be due to tumor tissue degeneration caused by tumor cell death. Comparatively, the largest morphological difference was observed in the NBP@mSiO_2_-PEG-cRGD/I3C-L group, with

cells displaying irregular shapes, shrunken nuclei, and loose arrangement, suggesting the best therapeutic effect. Furthermore, detection of apoptosis by terminal deoxynucleotidyl transferase-mediated dUTP nick end labeling (TUNEL) staining (Fig. 5G and S29) showed more red fluorescence in the NBP@mSiO_2_-PEG-cRGD/I3C-L group, suggesting that more tumor cells treated with NBP@mSiO_2_-PEG-cRGD/I3C under laser irradiation have undergone apoptosis. Correspondingly, Ki-67 immunofluorescence staining (Fig. 5H and S30) demonstrated that the NBP@mSiO_2_-PEG-cRGD/I3C-L group showed lowest cell proliferation biomarker Ki-67, indicating the treatment could significantly inhibit tumor cell proliferation.

In addition, studies have revealed that the dense micro-vessels of the tumor can supply sufficient oxygen and nutrients to the tumor tissue to promote its growth and metastasis [50]. As shown in Fig. 5I and S31, by performing immunofluorescence staining of CD31 (a vascular endothelial marker that is often used to assess angiogenesis in tumors), it can be clearly seen that tumor micro-vessels were the densest in the PBS group. In contrast, the NBP@mSiO_2_-PEG-cRGD/I3C-L group showed a significant reduction of tumor micro-vessels, likely due to the role of the efficient released I3C within tumors in inhibiting the formation of tumor micro-vessels [51]. Collectively, these results emphasized that the combination of tumor targeting, PTT, and photothermal-controlled release of I3C contributed to the satisfactory tumor-inhibitory effect conferred by only single-dose administration of NBP@mSiO_2_-PEG-cRGD/I3C when irradiated by laser, based on which the eradication of tumors can be expected if administration times increase.

### 3.6 Underlying mechanism of NBP@mSiO_2_-PEG-cRGD/I3C-based treatment

**Fig. 6:**
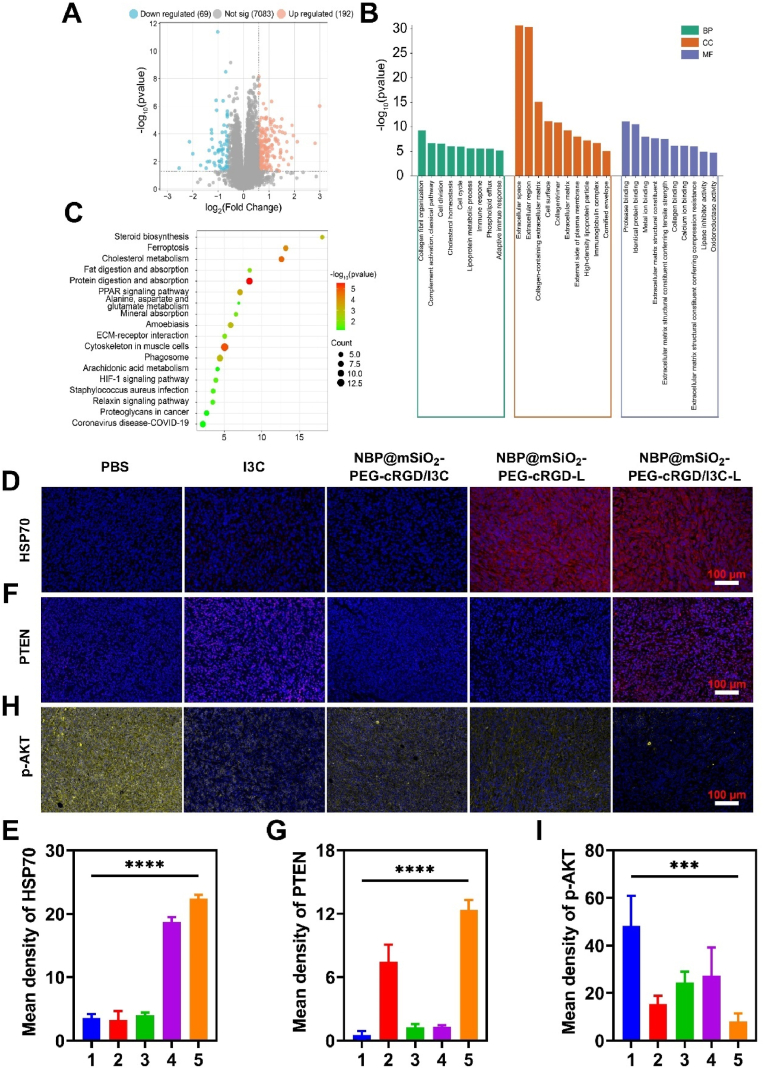
Anti-tumor mechanism of NBP@mSiO_2_-PEG-cRGD/I3C-based treatment. (A) Volcano plot for DEPs measured between the NBP@mSiO_2_-PEG-cRGD/I3C-L group and PBS group. (B) GO and (C) KEGG enrichment analyses of up-regulated proteins. (D) Immunofluorescence staining (Blue: DAPI for nucleus; Red: HSP70) of HSP70 within tumor tissues from different groups at the end of the treatment (Scale bar = 100 μm). (E) Quantitative analysis of the expression level of HSP70. (F) Immunofluorescence staining (Blue: DAPI for nucleus; Red: PTEN) of PTEN within tumor tissues from different treatment groups at the end of treatments (Scale bar = 100 μm). (G) Quantitative analysis of the expression level of PTEN. (H) Immunofluorescence staining (Blue: DAPI for nucleus; Yellow: p-AKT) of p-AKT within tumor tissues from different treatment groups at the end of treatments (Scale bar = 100 μm). (I) Quantitative analysis of the expression level of p-AKT. 1: PBS; 2: I3C; 3: NBP@mSiO_2_-PEG-cRGD/I3C; 4: NBP@mSiO_2_-PEG-cRGD-L; 5: NBP@mSiO_2_-PEG-cRGD/I3C-L; “L” represents laser irradiation. Data are expressed as the mean ± SD. Statistical analysis is performed using one-way ANOVA and Student's *t*-test. *∗∗∗p* < 0.001 and *∗∗∗∗p* < 0.0001 represent different statistical significances. (For interpretation of the references to color in this figure legend, the reader is referred to the Web version of this article.)

The above-verified effective treatment of NBP@mSiO_2_-PEG-cRGD/I3C under laser irradiation inspired us to explore the underlying mechanism. Proteomic analysis was firstly performed on tumor tissues of mice from the PBS group and NBP@mSiO_2_-PEG-cRGD/I3C-L group to characterize differentially expressed proteins (DEPs). Filtered by |log_2_ (fold change)| ≥ 1.5 and adjusted *p* value < 0.05, a total of 261 DEPs were identified, of which 192 proteins were significantly up-regulated and 69 proteins were significantly down-regulated (Fig. 6A). Furthermore, gene ontology (GO) enrichment analysis of up-regulated proteins was applied in terms of biological process (BP), molecular function (MF), and cellular composition (CC). And up-regulated proteins were mainly involved in the collagen fibril organization, extracellular space, collagen-containing extracellular matrix (ECM), protease binding, and identical protein binding (Fig. 6B). Besides, according to the kyoto encyclopedia of genes and genomes (KEGG) analysis, more up-regulated proteins were enriched into peroxisome proliferator-activated receptor (PPAR) and hypoxia-inducible factor-1 (HIF-1) signaling pathways commonly associated with tumor growth inhibition (Fig. 6C). The former triggered nuclear factor-kappa B (NF-κB) activation, cytokine secretion, and inflammatory responses. The activation of the inflammatory response further implied the occurrence of the PTT, echoing above *in vivo* photothermal conversion results (Fig. 4G and H) [52]. The latter exerted anti-tumor effects by activating effector immune cells and enhancing antigen presentation through up-regulated interferon gamma (IFN-γ) receptor 1. In addition, for the KEGG analysis of down-regulated proteins (Fig. S32), the ECM-receptor interaction, efferocytosis, and focal adhesion signaling pathways were primarily inhibited, all of which positively regulate cell growth. Among them, the inhibition of integrin beta 1 expression in the focal adhesion pathway can further suppress the downstream PI3K-AKT signaling pathway, echoing above results of photothermal-controlled release of I3C (Fig. 2G) [53].

According to proteomics results, we then investigated the key protein reflecting PTT, i.e., heat shock protein 70 (HSP70), as well as key PTEN and AKT proteins participating in the PI3K-AKT signaling pathway, through immunofluorescence staining, to consolidate the anti-tumor mechanism of NBP@mSiO_2_-PEG-cRGD/I3C-based treatment. HSP70 is a stress-inducible protein synthesized for self-protection when organisms are exposed to high temperatures [54,55]. As verified in Fig. 6D and E, the higher levels of HSP70 were observed in the laser irradiation groups (i.e., the NBP@mSiO_2_-PEG-cRGD-L and NBP@mSiO_2_-PEG-cRGD/I3C-L groups) compared to the non-laser irradiation groups (i.e., the PBS, I3C, and NBP@mSiO_2_-PEG-cRGD/I3C groups), evidencing the involvement of PTT in the anti-tumor process by NBP@mSiO_2_-PEG-cRGD/I3C-L, which was conferred by the efficient photothermal effects of NBP@mSiO_2_-PEG-cRGD.

The occurrence of PTT toward the tumor should contribute to the concomitant release of I3C from NBP@mSiO_2_-PEG-cRGD/I3C to regulate the expression of PTEN tumor suppressor protein, which should intervene in the PI3K-AKT signaling pathway according to recent findings to advance anti-tumor effect [21]. As shown in Fig. 6F and G, weak red fluorescence was observed in the PBS and NBP@mSiO_2_-PEG-cRGD-L groups, while stronger red fluorescence was revealed in the I3C group, indicating that the non-specific diffusion of I3C into tumor cells induced the expression of PTEN to a certain extent. Intriguingly, the NBP@mSiO_2_-PEG-cRGD/I3C group demonstrated insufficient PTEN protein expression due to the limited release of loaded I3C within tumor at the physiological environment of 37.0 °C as verified above (Fig. 6F and G). As expected, the highest expression level of PTEN was found in the NBP@mSiO_2_-PEG-cRGD/I3C-L group. The decent expression of PTEN induced by released I3C was expected to facilitate PTEN-mediated phosphatidylinositol-3,4,5-trisphosphate (PIP3) dephosphorylation to inhibit AKT phosphorylation (p-AKT), thus inhibiting the activation of PI3K-AKT signaling pathway. Indeed, as presented in Fig. 6H and I, the expression of p-AKT protein exhibited an opposite pattern to that of PTEN. Specifically, the PBS group displayed the highest expression level of p-AKT protein, with relatively lower levels of p-AKT detected in the NBP@mSiO_2_-PEG-cRGD/I3C and NBP@mSiO_2_-PEG-cRGD-L groups, while nearly no p-AKT protein can be detected in the I3C and NBP@mSiO_2_-PEG-cRGD/I3C-L group. Collectively, all these data confirmed the elicitation of chemo-photothermal combinatory therapy by NBP@mSiO_2_-PEG-cRGD/I3C once irradiated by NIR laser, thus achieving the efficient inhibition of breast tumors.

## 4 Conclusion

In summary, a multifunctional nanoplatform (NBP@mSiO_2_-PEG-cRGD) combining advances of tumor targeting, superior biocompatibility, excellent photothermal effect, high loading capacity of I3C, and photothermal-controlled I3C release was successfully designed and constructed. *In vitro* experiments showed that NBP@mSiO_2_-PEG-cRGD/I3C could effectively facilitate cellular uptake as well as inhibit cell proliferation and promote cell apoptosis under laser irradiation. Specifically, the proliferation inhibition rate of 4T1 and MDA-MB-231 cells conferred by NBP@mSiO_2_-PEG-cRGD/I3C under laser irradiation reached as high as 92.6 % and 90.8 %, respectively. *In vivo* experiments demonstrated that only single-dose administration of biocompatible NBP@mSiO_2_-PEG-cRGD/I3C under laser irradiation guided by fluorescence imaging exhibited a superior therapeutic effect, manifesting in significant inhibition of 4T1 tumor growth. The underlying therapeutic mechanism was deciphered as the efficient photothermal conversion of NBP@mSiO_2_-PEG-cRGD under irradiation simultaneously induced PTT to combat the tumor and promoted the release of loaded I3C, of which the later inhibited the phosphorylation of AKT by up-regulating the expression of PTEN to inhibit the activation of PI3K-AKT signaling pathway. Such process led to controlled chemo-photothermal combinational therapeutic mode, achieving the effective combat of breast cancer. By virtue of the customization of loaded anti-tumor agents and indiscriminate tumor cell killing ability of PTT, the nanoplatform we developed here can be generalized to deal with a broad spectrum of cancer.

## CRediT authorship contribution statement

**Lin Yang:** Writing – original draft, Software, Methodology, Investigation, Formal analysis, Data curation, Conceptualization. **Ying Zhang:** Writing – review & editing, Supervision, Software. **Jing Liu:** Software, Methodology. **Xiaofen Wang:** Writing – review & editing. **Li Zhang:** Data curation. **Hao Wan:** Writing – review & editing, Visualization, Validation, Supervision, Project administration, Funding acquisition, Conceptualization.

## Declaration of competing interest

The authors declare that they have no known competing financial interests or personal relationships that could have appeared to influence the work reported in this paper.

## Data Availability

Data will be made available on request.
