# Supplementary material for: A tumor-targeting black phosphorus-based nanoplatform for controlled chemo-photothermal therapy of breast cancer
Source: Mater Today Bio. 2025 Feb 7;31:101563. doi: 10.1016/j.mtbio.2025.101563 (PMC11870200; doi:10.1016/j.mtbio.2025.101563)
Supplement: Multimedia component 1 [file mmc1.docx]

**Supplementary Material**

**A tumor-targeting black phosphorus-based nanoplatform for** **controlled chemo-photothermal therapy of breast cancer**

Lin Yang^1^, Ying Zhang^1^, Jing Liu^2^, Xiaofen Wang^3^, Li Zhang^1^, and Hao Wan^1^*

^1^State Key Laboratory of Food Science and Resources, Nanchang University, Nanchang 330047, P. R. China

^2^College of Pharmacy, Dalian Medical University, Dalian, 116044, P. R. China.

^3^Cancer Research Center, Jiangxi University of Chinese Medicine, Nanchang 330004,

*Corresponding author: wanhao424@ncu.edu.cn

These authors contributed to the work equally: L. Yang, Y. Zhang, and J. Liu

**Contents**

**Fig. S1.** (A) The UV-VIS spectrum and (B) the standard curve of I3C.

**Fig. S2.** XPS of NBP@mSiO_2_-PEG-cRGD.

**Fig. S3.** SEM image of NBP.

**Fig. S4.** SEM image of individual NBP.

**Fig. S5.** SEM image of NBP@mSiO_2_-PEG-cRGD.

**Fig. S6.** AFM image of NBP.

**Fig. S7.** Zeta potential changes of NBP@mSiO_2_-PEG-cRGD in PBS (pH = 7.4) over 7 days.

**Fig. S8.** The photothermal images of different material solutions with an equivalent concentration of BP under 808 nm laser irradiation of 2 W/cm^2^ at various time points.

**Fig. S9.** Temperature change of NBP and NBP@mSiO_2_ solution post one-week storage over time under 808 nm laser irradiation at 2 W/cm^2^.

**Fig. S10.** The photothermal images of NBP@mSiO_2_-PEG-cRGD with different concentrations under 808 nm laser irradiation of 2 W/cm^2^ at various time points.

**Fig. S11.** Photothermal stability of NBP@mSiO_2_-PEG-cRGD (100 μg/mL), with PBS acting as the control comparison.

**Fig. S12.** Fluorescence intensity quantification for phagocytosis experiments of 4T1 cells and HEK293T cells.

**Fig. S13.** Viabilities of MCF-7 cells treated with different concentrations of NBP@mSiO_2_-PEG-cRGD for 3 h, 6 h, and 12 h, respectively.

**Fig. S14.** Viabilities of HEK293T cells treated with different concentrations of NBP@mSiO_2_-PEG-cRGD for 3 h, 6 h, and 12 h, respectively.

**Fig. S15.** Viabilities of 4T1 cells under different treatment scenarios (i.e., PBS, I3C (1.38 μg/mL), NBP@mSiO_2_-PEG-cRGD/I3C (NBP@mSiO_2_-PEG-cRGD: 25 μg/mL), NBP@mSiO_2_-PEG-cRGD-L (NBP@mSiO_2_-PEG-cRGD: 25 μg/mL), and NBP@mSiO_2_-PEG-cRGD/I3C-L (NBP@mSiO_2_-PEG-cRGD: 25 μg/mL); “L” represents “808 nm laser irradiation at 2 W/cm^2^ for 10 min”).

**Fig. S16.** Viabilities of 4T1 cells under different treatment scenarios (i.e., PBS, I3C (2.75 μg/mL), NBP@mSiO_2_-PEG-cRGD/I3C (NBP@mSiO_2_-PEG-cRGD: 50 μg/mL), NBP@mSiO_2_-PEG-cRGD-L (NBP@mSiO_2_-PEG-cRGD: 50 μg/mL), and NBP@mSiO_2_-PEG-cRGD/I3C-L (NBP@mSiO_2_-PEG-cRGD: 50 μg/mL); “L” represents “808 nm laser irradiation at 2 W/cm^2^ for 10 min”).

**Fig. S17.** Viabilities of MDA-MB-231 cells under different treatment scenarios (i.e., PBS, I3C (1.38 μg/mL), NBP@mSiO_2_-PEG-cRGD/I3C (NBP@mSiO_2_-PEG-cRGD: 25 μg/mL), NBP@mSiO_2_-PEG-cRGD-L (NBP@mSiO_2_-PEG-cRGD: 25 μg/mL), and NBP@mSiO_2_-PEG-cRGD/I3C-L (NBP@mSiO_2_-PEG-cRGD: 25 μg/mL); “L” represents “808 nm laser irradiation at 2 W/cm^2^ for 10 min”).

**Fig. S18.** Viabilities of MDA-MB-231 cells under different treatment scenarios (i.e., PBS, I3C (2.75 μg/mL), NBP@mSiO_2_-PEG-cRGD/I3C (NBP@mSiO_2_-PEG-cRGD: 50 μg/mL), NBP@mSiO_2_-PEG-cRGD-L (NBP@mSiO_2_-PEG-cRGD: 50 μg/mL), and NBP@mSiO_2_-PEG-cRGD/I3C-L (NBP@mSiO_2_-PEG-cRGD: 50 μg/mL); “L” represents “808 nm laser irradiation at 2 W/cm^2^ for 10 min”).

**Fig. S19.** Fluorescence intensity quantification of live/dead staining of 4T1 cells treated with different scenarios.

**Fig. S20.** Live/dead staining of MDA-MB-231 cells treated with different scenarios.

**Fig. S21.** Fluorescence intensity quantification of MMP variation of 4T1 cells treated with different scenarios.

**Fig. S22.** MMP variation characterized by JC-1 staining after treating MDA-MB-231 cells with different scenarios.

**Fig. S23.** Hemolysis of NBP@mSiO_2_-PEG-cRGD at various concentrations.

**Fig. S24.** ICG fluorescence spectrum.

**Fig. S25.** Immunohistochemical staining of αvβ3 integrin in the 4T1 breast tumor tissues and normal breast tissues.

**Fig. S26.** *In vivo* fluorescence imaging of 4T1 tumor-bearing mice after intravenous injection of Cy5-labeled NBP@mSiO_2_-PEG-MAL (Cy5-NBP@mSiO_2_-PEG-MAL) or Cy5-labeled NBP@mSiO_2_-PEG-cRGD (Cy5-NBP@mSiO_2_-PEG-cRGD).

**Fig. S27.** Viabilities of 4T1 cells under different treatment scenarios (i.e., NBP@mSiO_2_-PEG-MAL/I3C-L and NBP@mSiO_2_-PEG-cRGD/I3C-L; “L” represents “808 nm laser irradiation at 2 W/cm^2^ for 10 min”).

**Fig. S28.** Organ indices (i.e., heart index, liver index, spleen index, lung index, and kidney index) in mice after different treatments.

**Fig. S29.** Mean fluorescence intensity of TUNEL staining.

**Fig. S30.** Mean fluorescence intensity of Ki-67 staining.

**Fig. S31.** Mean fluorescence intensity of CD31 staining.

**Fig. S32.** KEGG enrichment analysis of down-regulated protein.


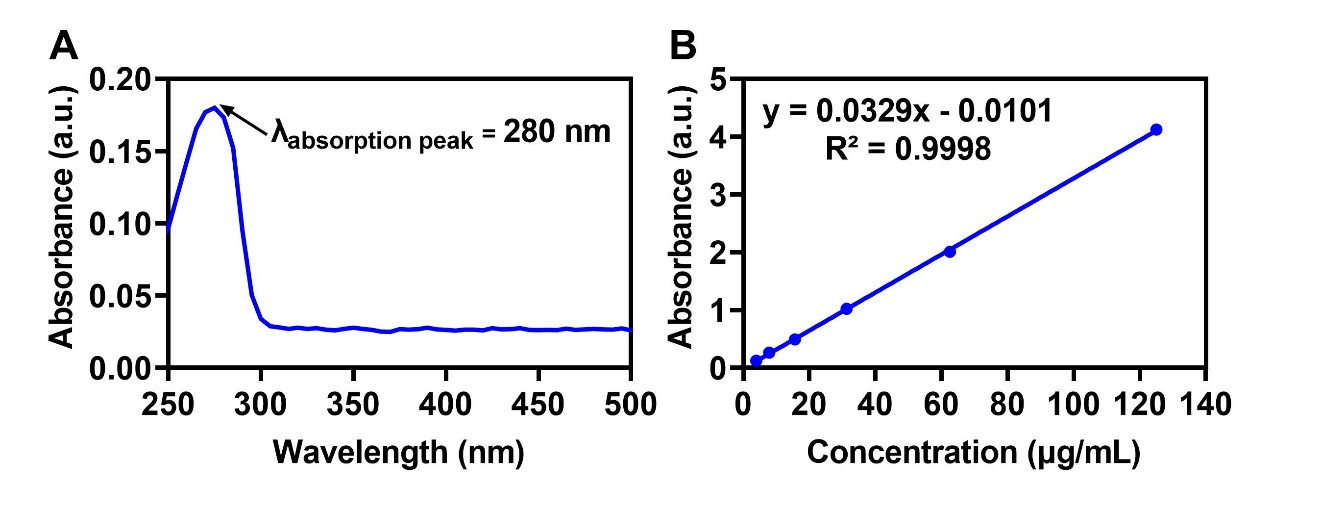


**Fig. S1.** (A) The UV-VIS spectrum and (B) the standard curve of I3C.


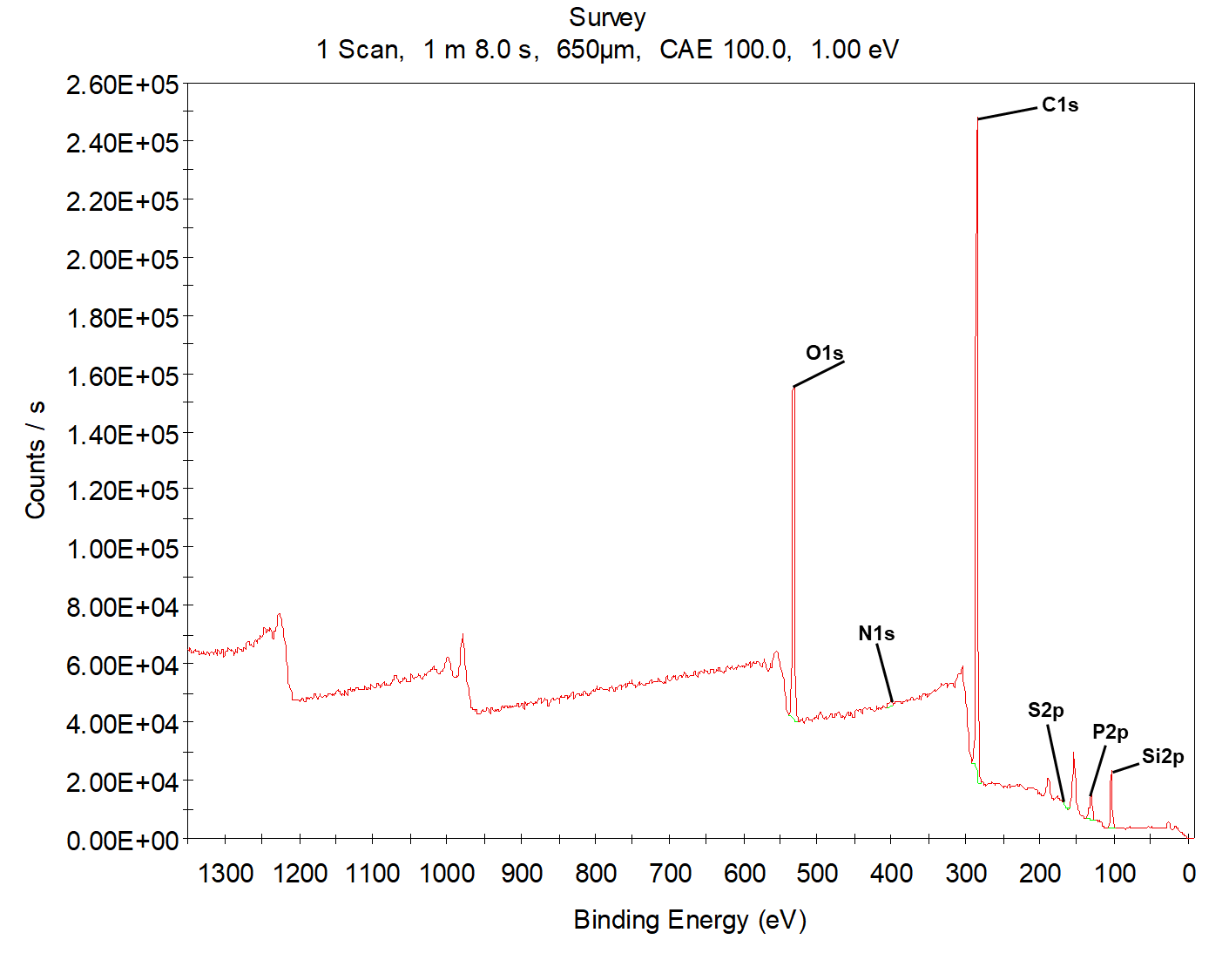


**Fig. S2.** XPS of NBP@mSiO_2_-PEG-cRGD.


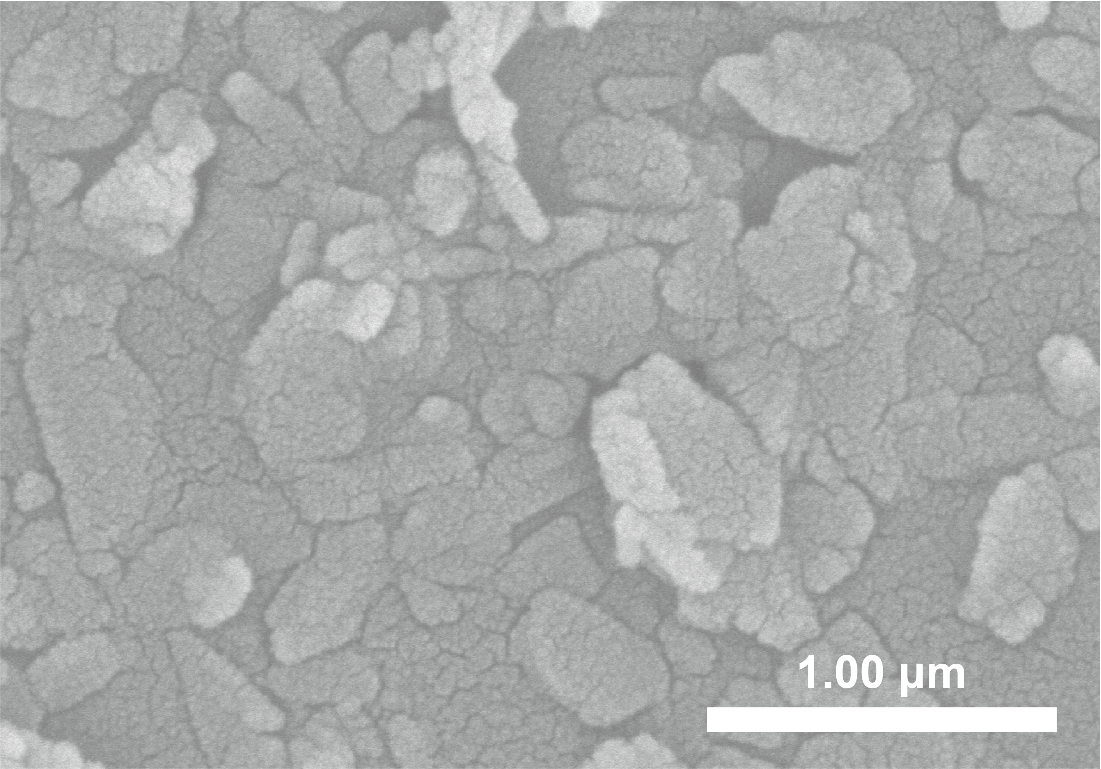


**Fig. S3.** SEM image of NBP. Scale bar = 1 μm.


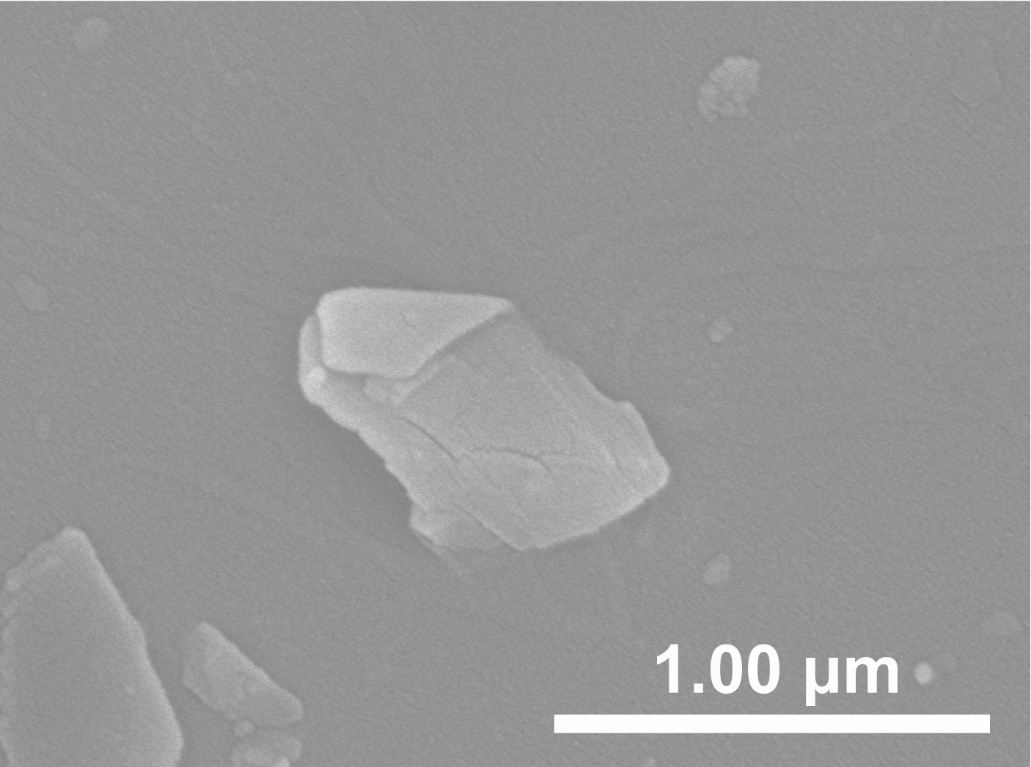


**Fig. S4.** SEM image of individual NBP. Scale bar = 1 μm.


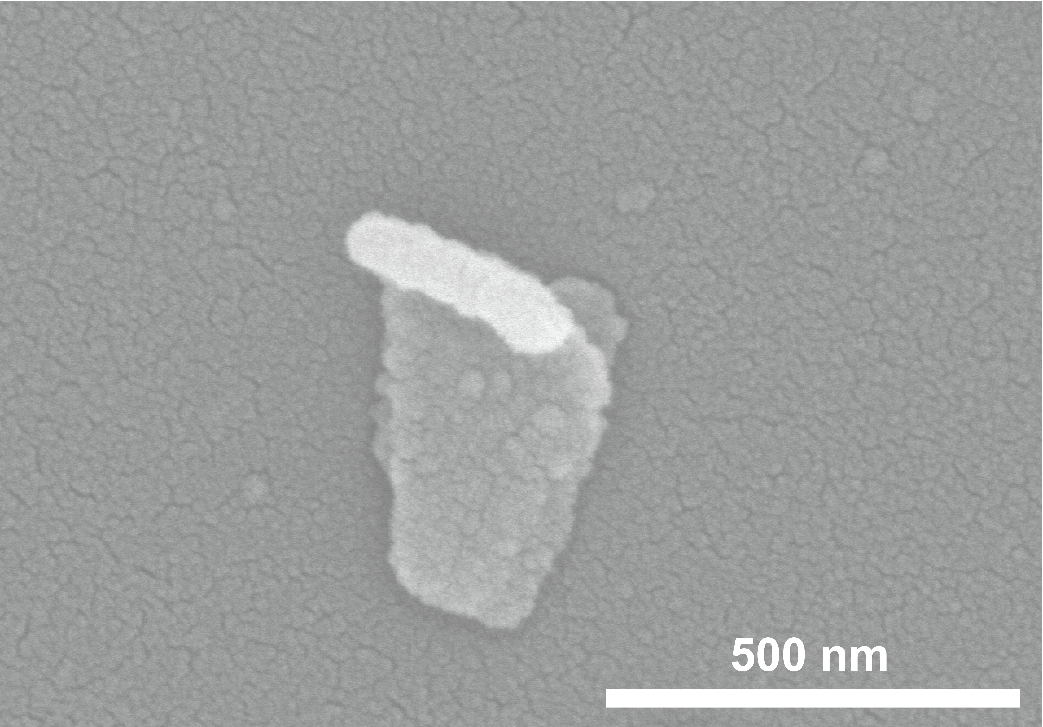


**Fig. S5.** SEM image of NBP@mSiO_2_-PEG-cRGD. Scale bar = 500 nm.


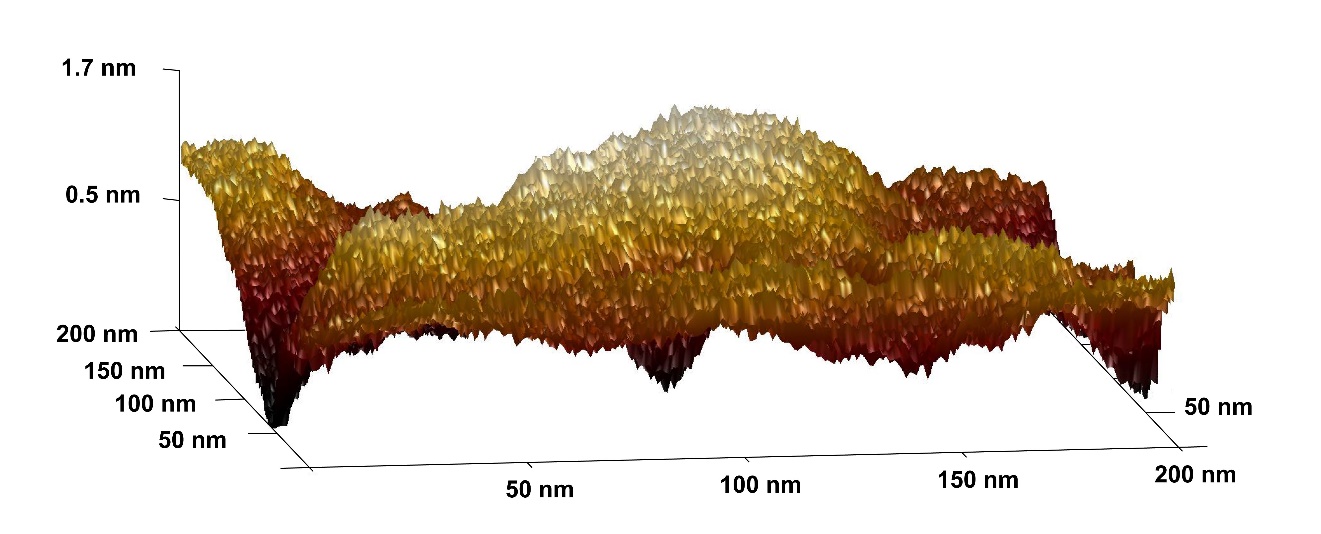


**Fig. S6.** AFM image of NBP.


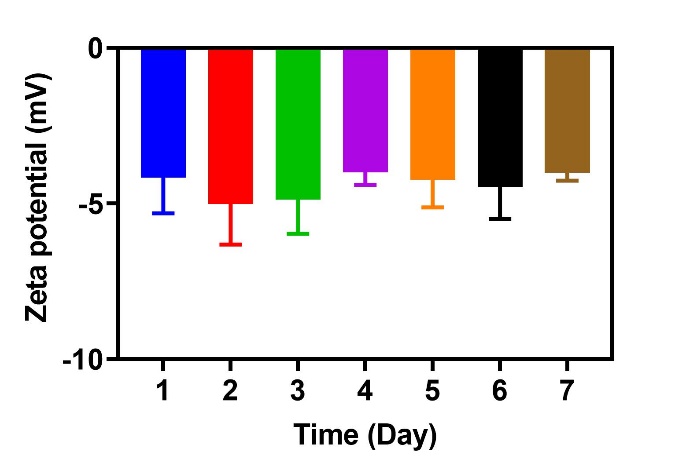


**Fig. S7.** Zeta potential changes of NBP@mSiO_2_-PEG-cRGD in PBS (pH = 7.4) over 7 days (n = 3). Data are expressed as the mean ± SD.


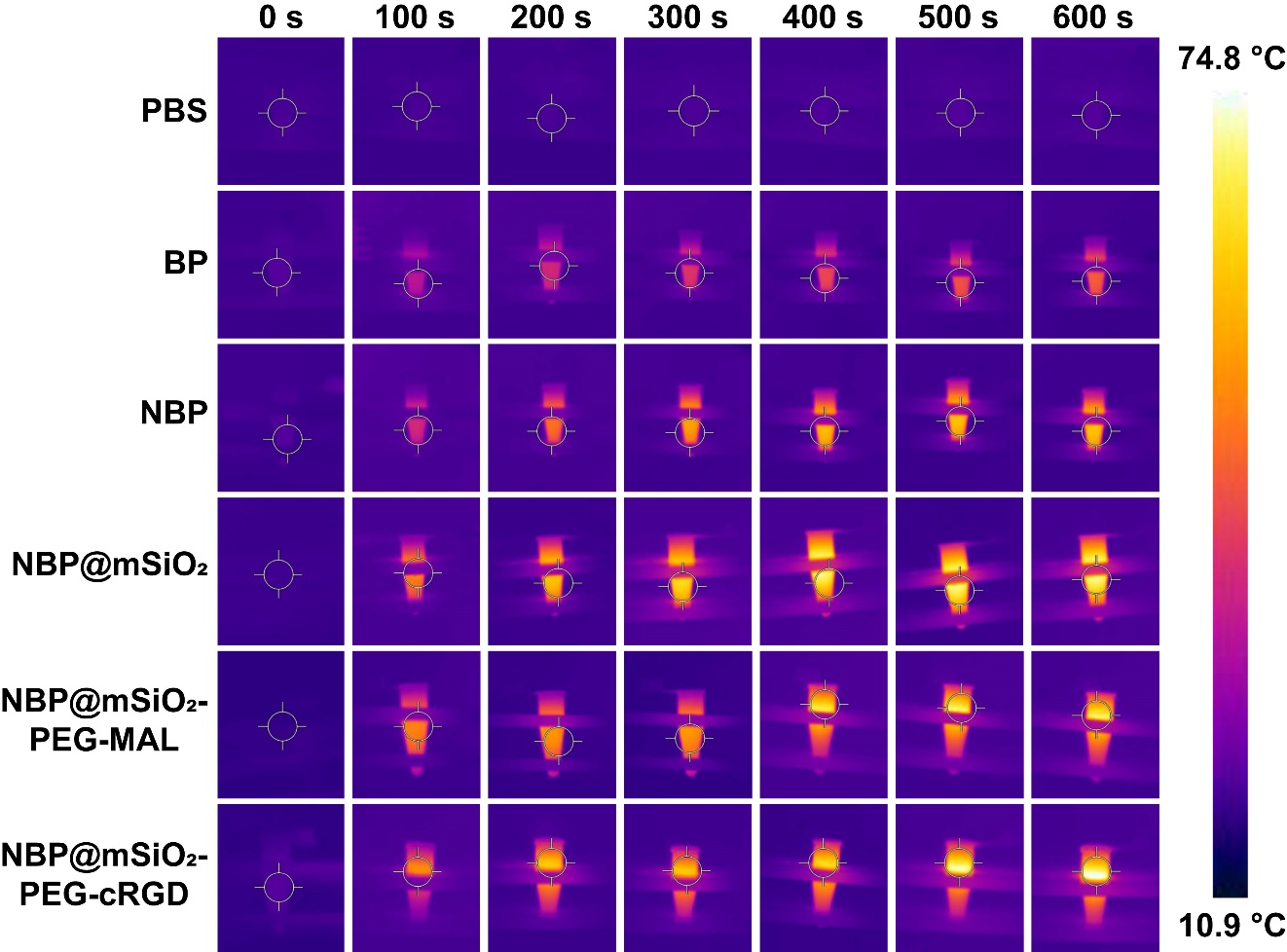


**Fig. S8.** The photothermal images of different material solutions with an equivalent concentration of BP under 808 nm laser irradiation of 2 W/cm² at various time points.


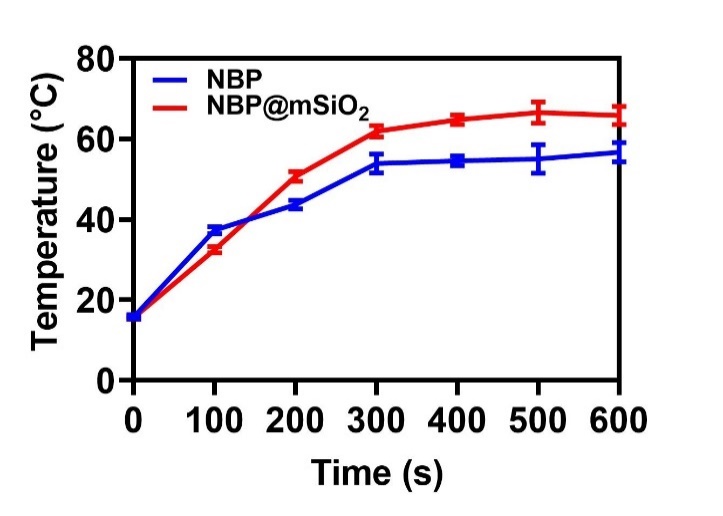


**Fig. S9.** Temperature change of NBP and NBP@mSiO_2_ solution post one-week storage over time under 808 nm laser irradiation at 2 W/cm². Data are expressed as the mean ± SD.

**
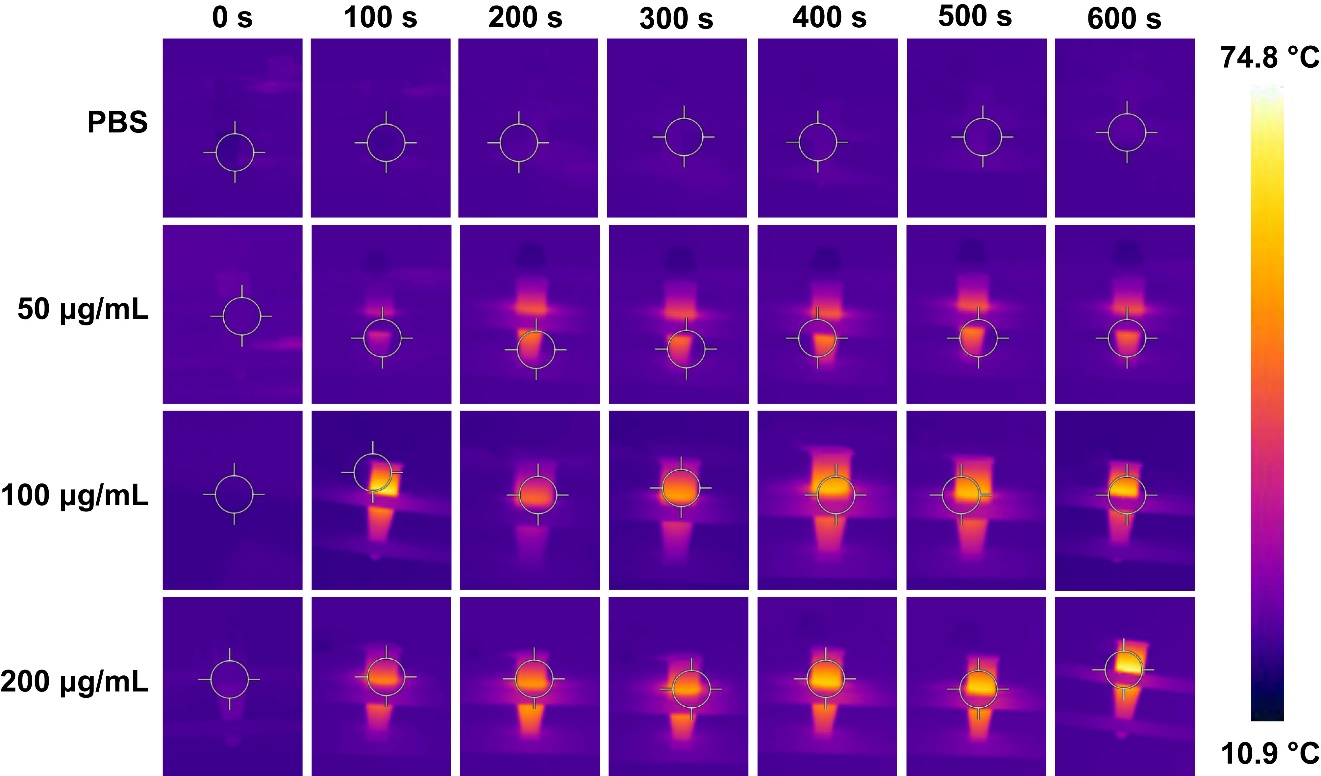
**

**Fig. S10.** The photothermal images of NBP@mSiO_2_-PEG-cRGD with different concentrations (i.e., 0, 50, 100, and 200 μg/mL) under 808 nm laser irradiation of 2 W/cm² at various time points.


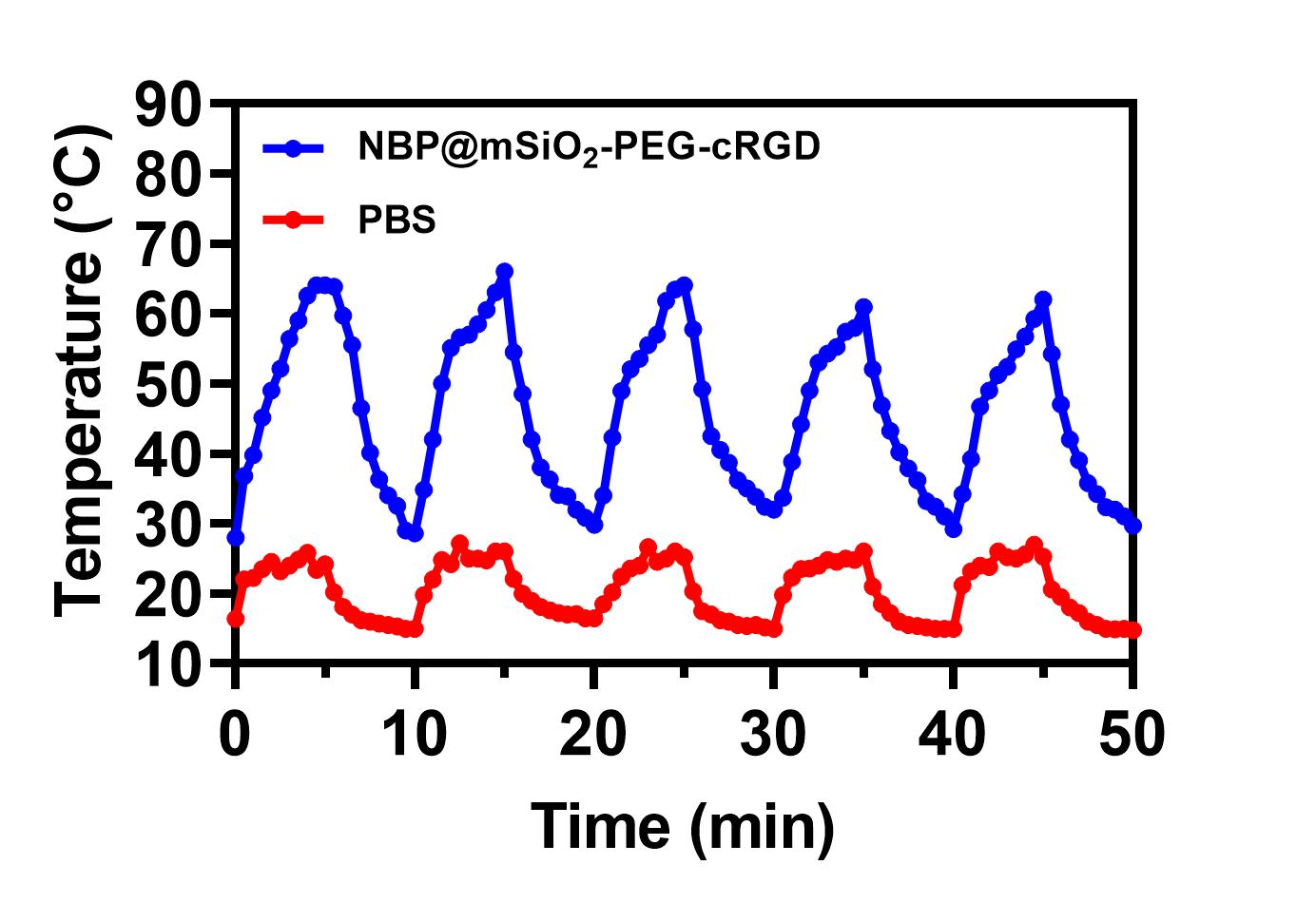


**Fig. S11.** Photothermal stability of NBP@mSiO_2_-PEG-cRGD (100 μg/mL), with PBS acting as the control comparison.


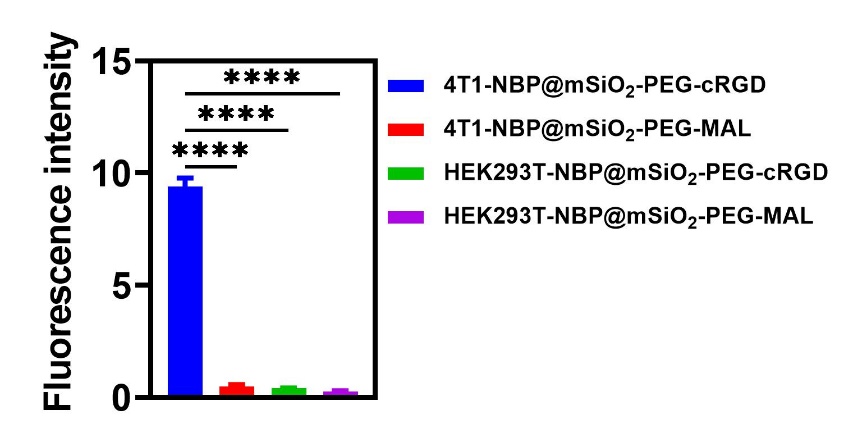


**Fig. S12.** Fluorescence intensity quantification for phagocytosis experiments of 4T1 cells and HEK293T cells toward various formulations. Data are expressed as the mean ± SD. Statistical analysis is performed using one-way ANOVA and Student’s *t* test. *****p* < 0.0001 represents a statistical significance.


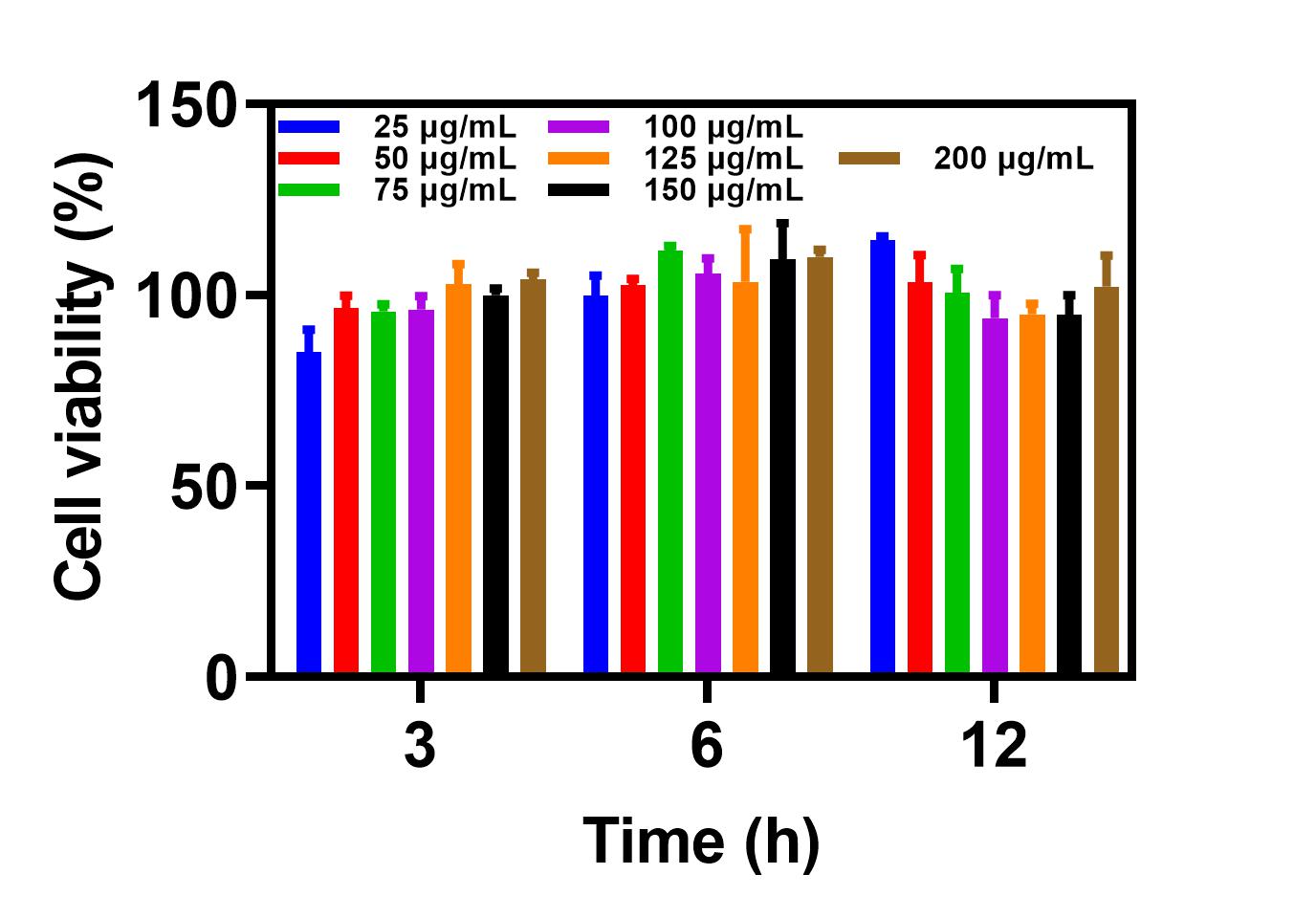


**Fig. S13.** Viabilities of MCF-7 cells treated with different concentrations of NBP@mSiO_2_-PEG-cRGD for 3 h, 6 h, and 12 h, respectively.


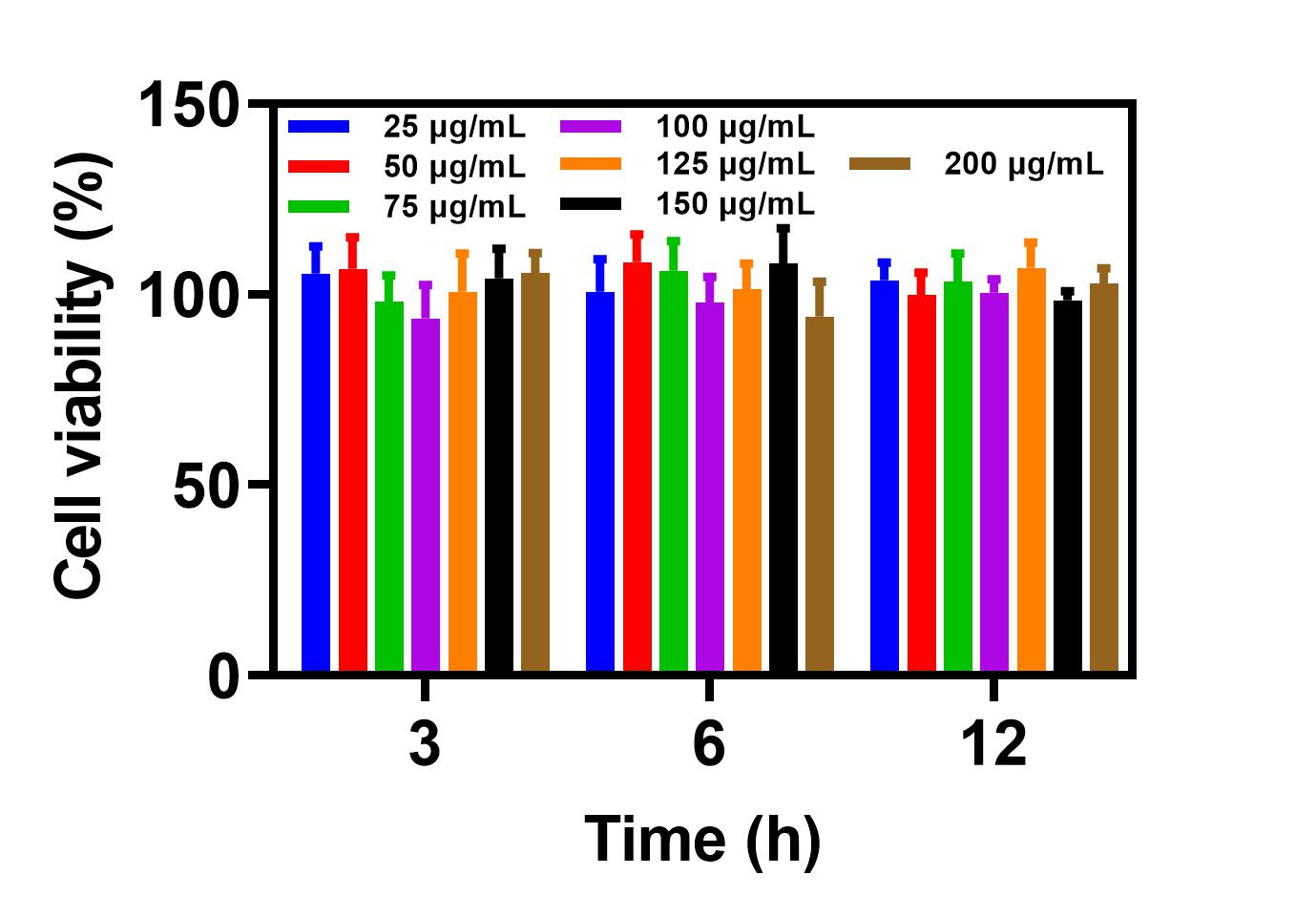


**Fig. S14.** Viabilities of HEK293T cells treated with different concentrations of NBP@mSiO_2_-PEG-cRGD for 3 h, 6 h, and 12 h, respectively.


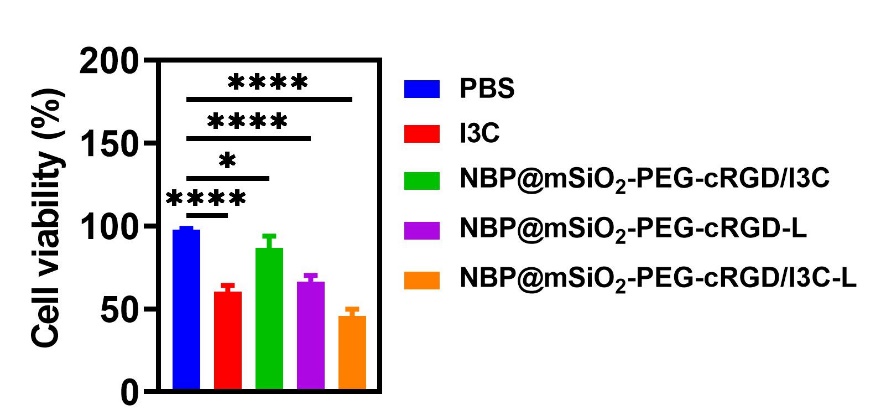


**Fig. S15.** Viabilities of 4T1 cells under different treatment scenarios (i.e., PBS, I3C (1.38 μg/mL), NBP@mSiO_2_-PEG-cRGD/I3C (NBP@mSiO_2_-PEG-cRGD: 25 μg/mL), NBP@mSiO_2_-PEG-cRGD-L (NBP@mSiO_2_-PEG-cRGD: 25 μg/mL), and NBP@mSiO_2_-PEG-cRGD/I3C-L (NBP@mSiO_2_-PEG-cRGD: 25 μg/mL); “L” represents “808 nm laser irradiation at 2 W/cm^2^ for 10 min”). Data are expressed as the mean ± SD. Statistical analysis is performed using one-way ANOVA and Student’s *t* test. **p* < 0.05 and *****p* < 0.0001 represent different statistical significances.


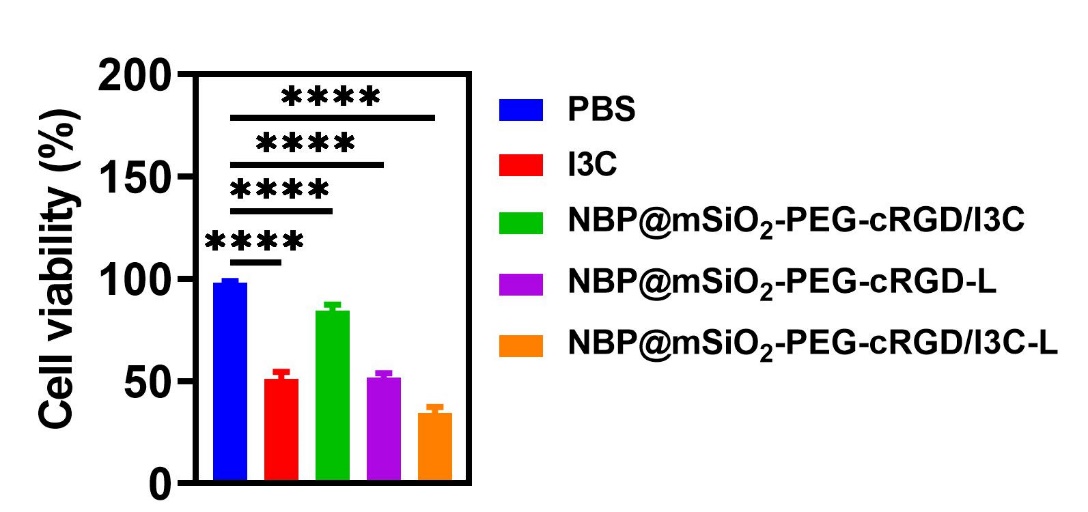


**Fig. S16.** Viabilities of 4T1 cells under different treatment scenarios (i.e., PBS, I3C (2.75 μg/mL), NBP@mSiO_2_-PEG-cRGD/I3C (NBP@mSiO_2_-PEG-cRGD: 50 μg/mL), NBP@mSiO_2_-PEG-cRGD-L (NBP@mSiO_2_-PEG-cRGD: 50 μg/mL), and NBP@mSiO_2_-PEG-cRGD/I3C-L (NBP@mSiO_2_-PEG-cRGD: 50 μg/mL); “L” represents “808 nm laser irradiation at 2 W/cm^2^ for 10 min”). Data are expressed as the mean ± SD. Statistical analysis is performed using one-way ANOVA and Student’s *t* test. *****p* < 0.0001 represents a statistical significance.


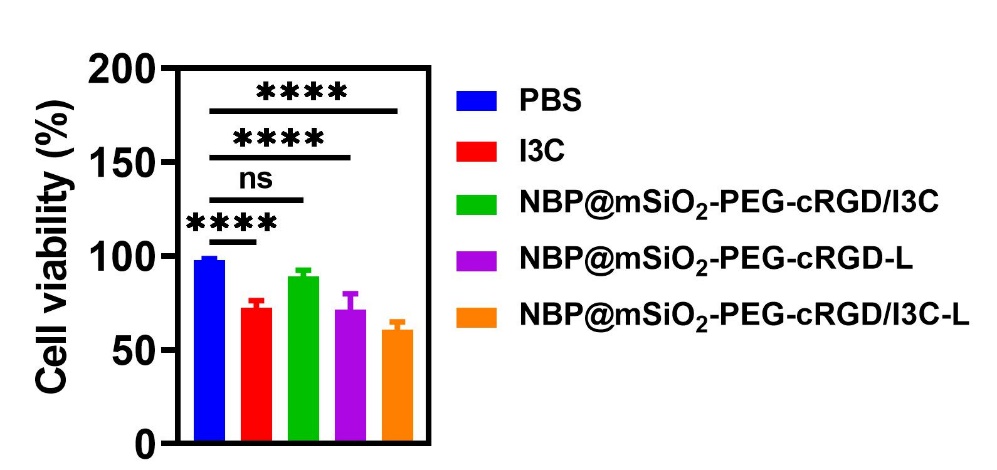


**Fig. S17.** Viabilities of MDA-MB-231 cells under different treatment scenarios (i.e., PBS, I3C (1.38 μg/mL), NBP@mSiO_2_-PEG-cRGD/I3C (NBP@mSiO_2_-PEG-cRGD: 25 μg/mL), NBP@mSiO_2_-PEG-cRGD-L (NBP@mSiO_2_-PEG-cRGD: 25 μg/mL), and NBP@mSiO_2_-PEG-cRGD/I3C-L (NBP@mSiO_2_-PEG-cRGD: 25 μg/mL); “L” represents “808 nm laser irradiation at 2 W/cm^2^ for 10 min”). Data are expressed as the mean ± SD. Statistical analysis is performed using one-way ANOVA and Student’s *t* test. *****p* < 0.0001 represents a statistical significance. “ns” means no significant difference.


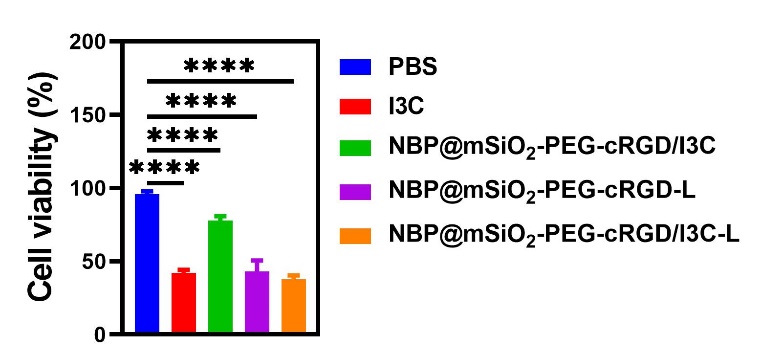


**Fig. S18.** Viabilities of MDA-MB-231 cells under different treatment scenarios (i.e., PBS, I3C (2.75 μg/mL), NBP@mSiO_2_-PEG-cRGD/I3C (NBP@mSiO_2_-PEG-cRGD: 50 μg/mL), NBP@mSiO_2_-PEG-cRGD-L (NBP@mSiO_2_-PEG-cRGD: 50 μg/mL), and NBP@mSiO_2_-PEG-cRGD/I3C-L (NBP@mSiO_2_-PEG-cRGD: 50 μg/mL); “L” represents “808 nm laser irradiation at 2 W/cm^2^ for 10 min”). Data are expressed as the mean ± SD. Statistical analysis is performed using one-way ANOVA and Student’s *t* test. *****p* < 0.0001 represents a statistical significance.


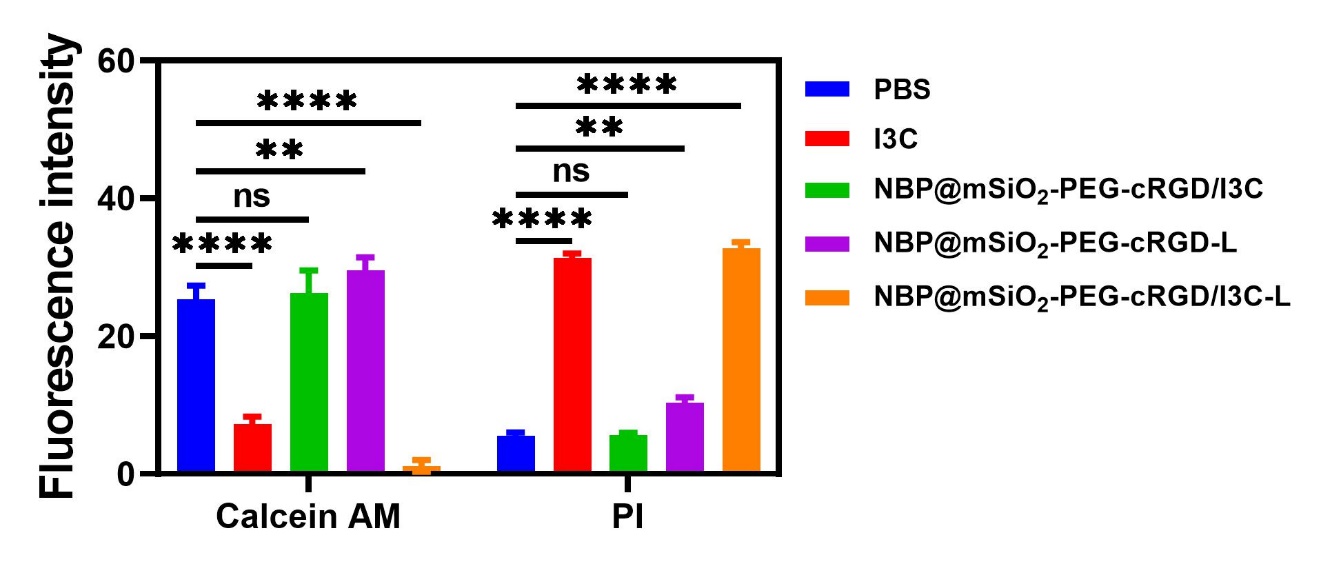


**Fig. S19.** Fluorescence intensity quantification of live/dead staining of 4T1 cells treated with different scenarios. Data are expressed as the mean ± SD. Statistical analysis is performed using two-way ANOVA and Student’s *t* test. ***p* < 0.01 and *****p* < 0.0001 represent different statistical significances. “ns” means no significant difference.


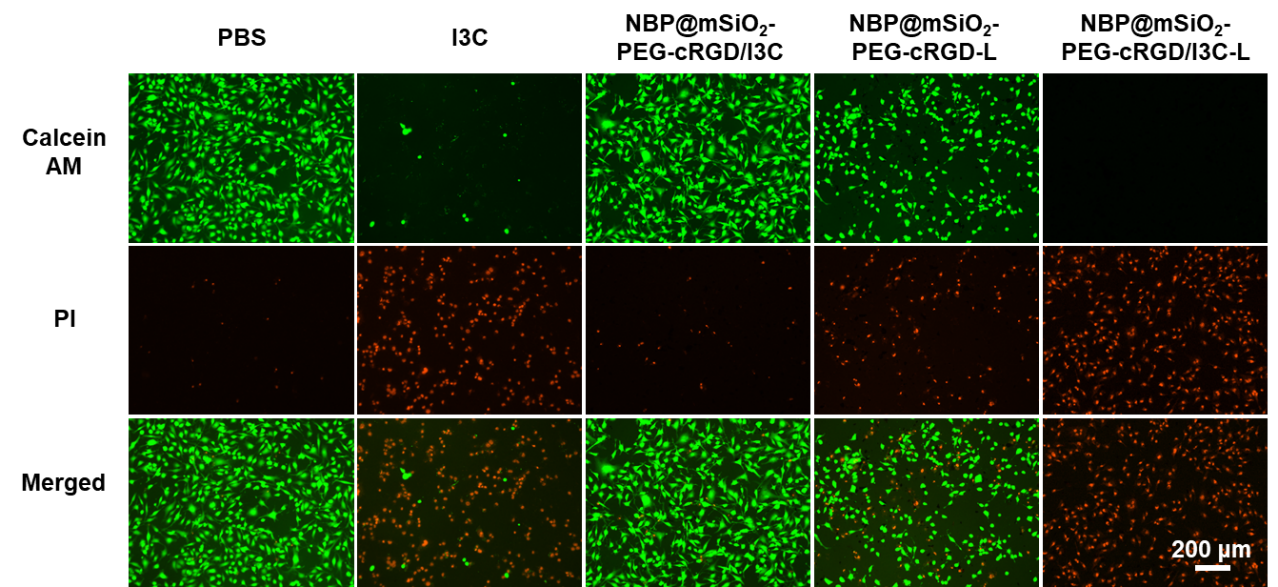


**Fig. S20.** Live/dead staining of MDA-MB-231 cells treated with different scenarios (i.e., PBS, I3C, NBP@mSiO_2_-PEG-cRGD/I3C, NBP@mSiO_2_-PEG-cRGD-L, and NBP@mSiO_2_-PEG-cRGD/I3C-L; “L” represents “laser irradiation”); Green: living cells were stained with Calcein-AM, Red: dead cells were stained with PI; Scale bar = 200 μm.


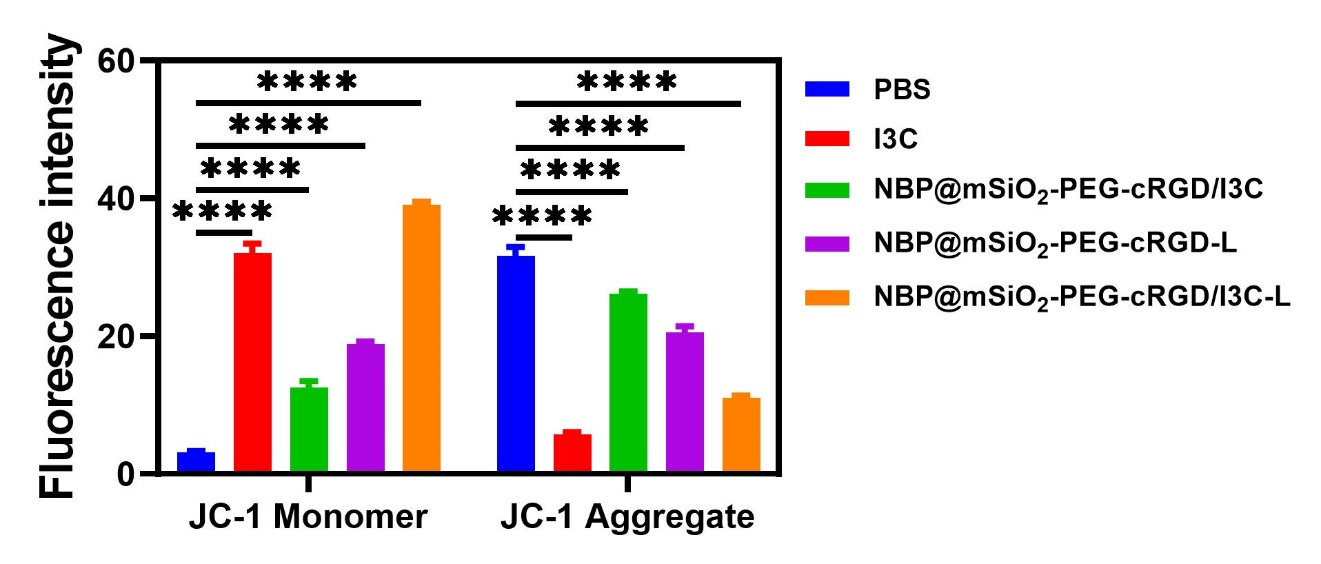


**Fig. S21.** Fluorescence intensity quantification of MMP variation of 4T1 cells treated with different scenarios. Data are expressed as the mean ± SD. Statistical analysis is performed using two-way ANOVA and Student’s *t* test. *****p* < 0.0001 represents a statistical significance.


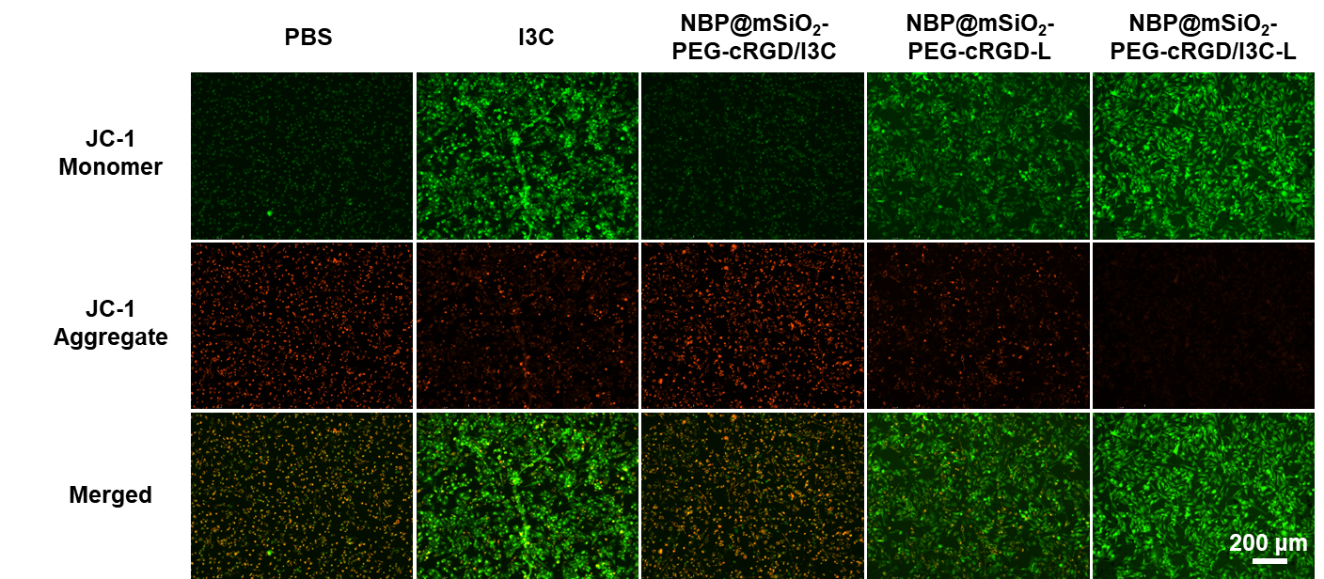


**Fig. S22.** MMP variation characterized by JC-1 staining after treating MDA-MB-231 cells with different scenarios (i.e., PBS, I3C, NBP@mSiO_2_-PEG-cRGD/I3C, NBP@mSiO_2_-PEG-cRGD-L, and NBP@mSiO_2_-PEG-cRGD/I3C-L; “L” represents “laser irradiation”; Red: JC-1 aggregate, Green: JC-1 monomer). Scale bar = 200 μm.


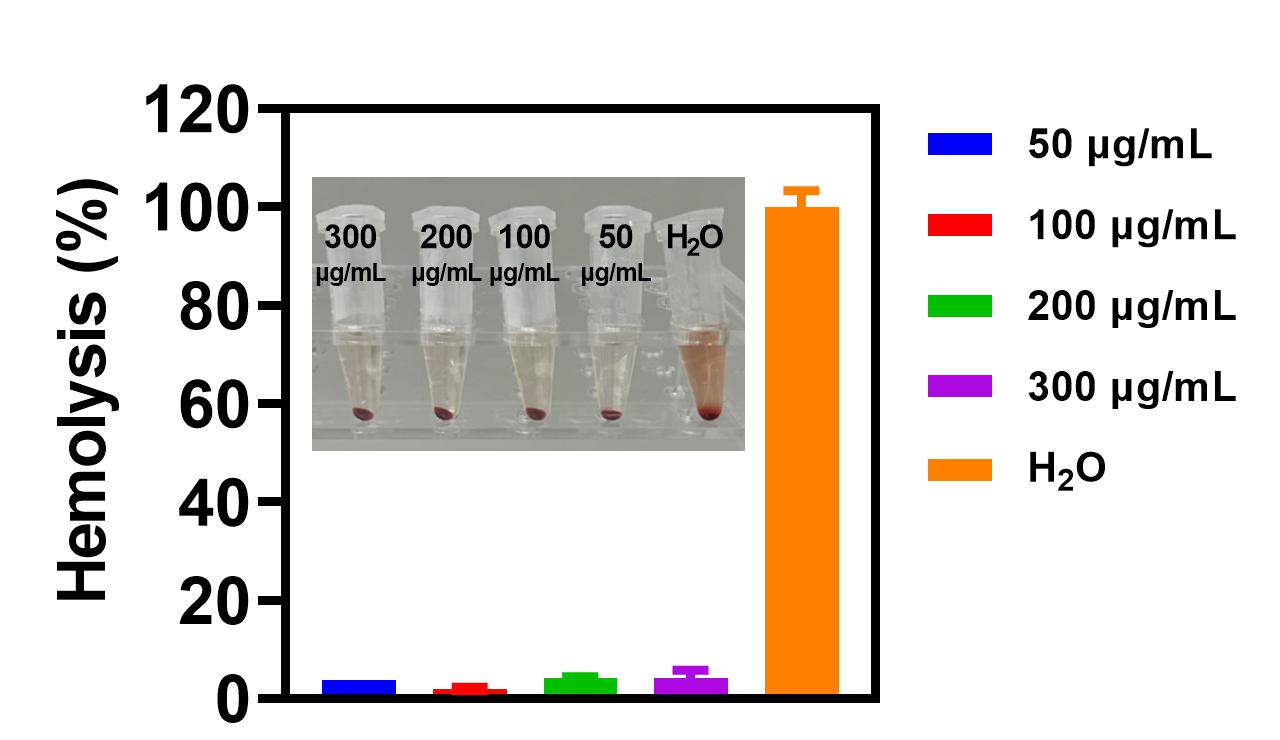


**Fig. S23.** Hemolysis of NBP@mSiO_2_-PEG-cRGD at various concentrations.


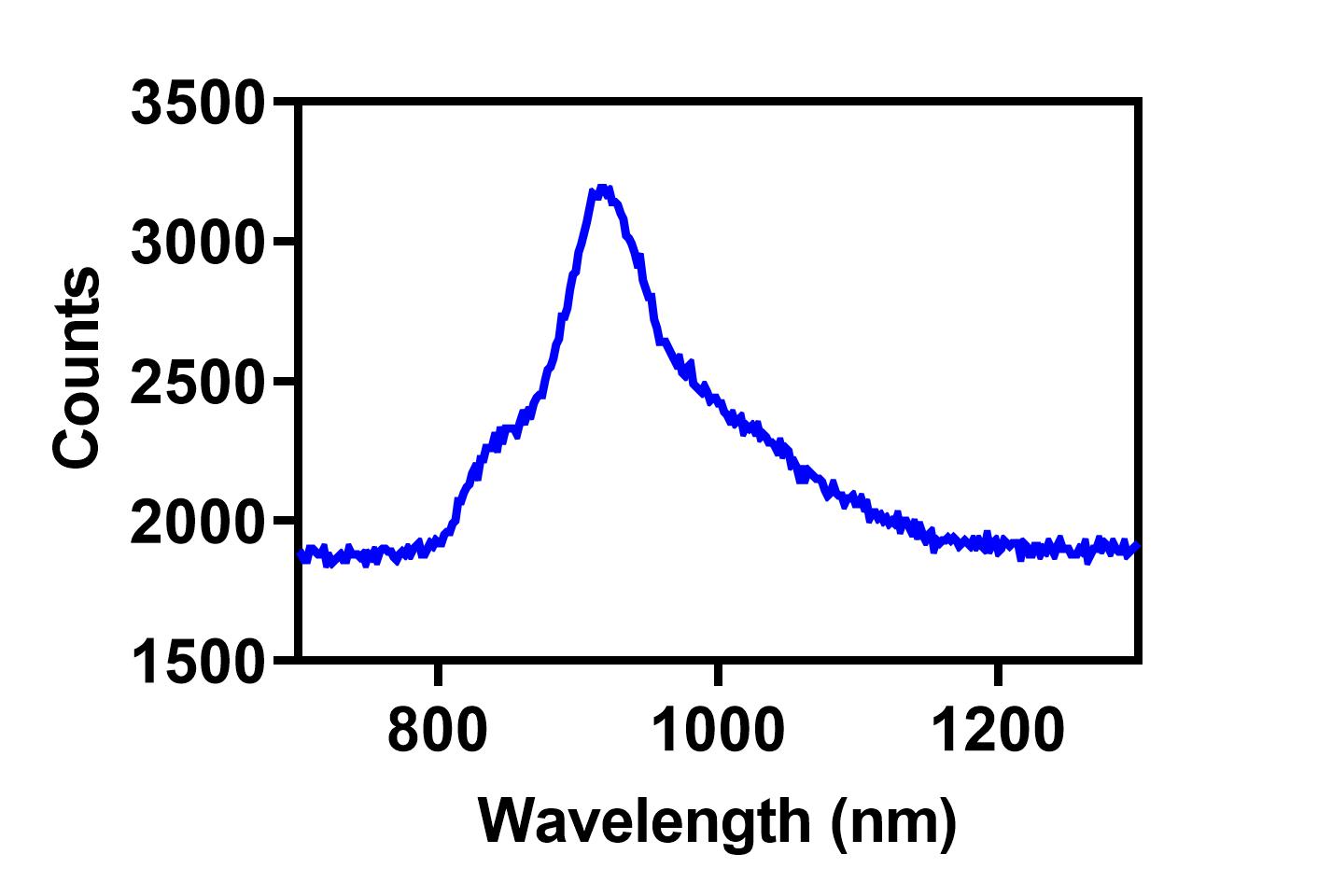


**Fig. S24.** ICG fluorescence spectrum.


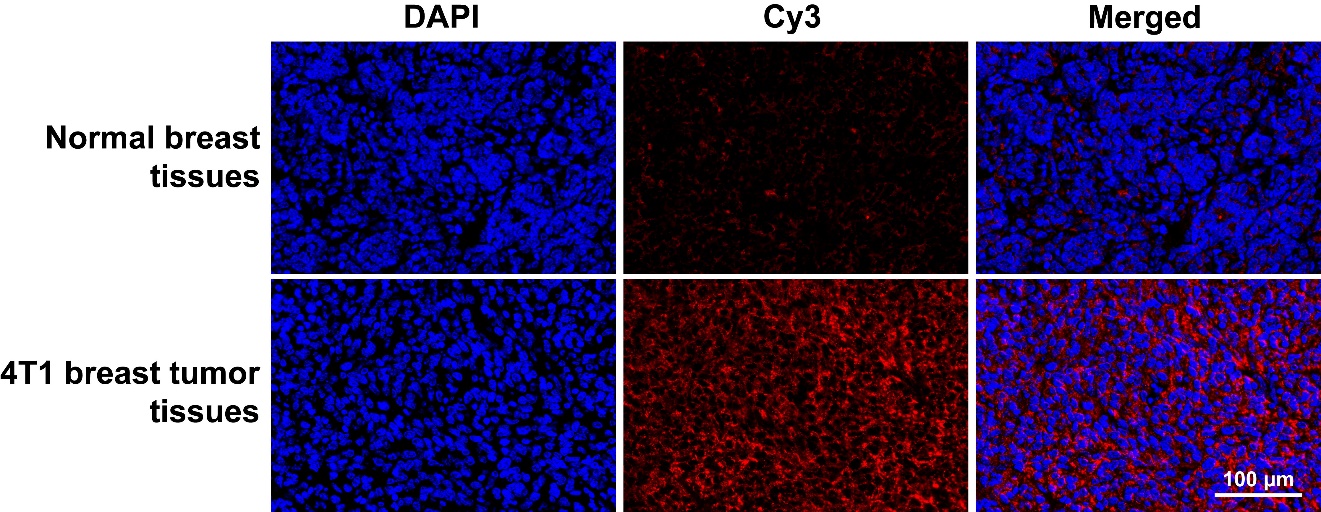


**Fig. S25.** Immunohistochemical staining of αvβ3 integrin in the 4T1 breast tumor tissues and normal breast tissues. Blue: Nucleus was stained by DAPI; Red: αvβ3 integrin was labeled by Cy3-anti-αvβ3 integrin. Scale bar = 100 μm.


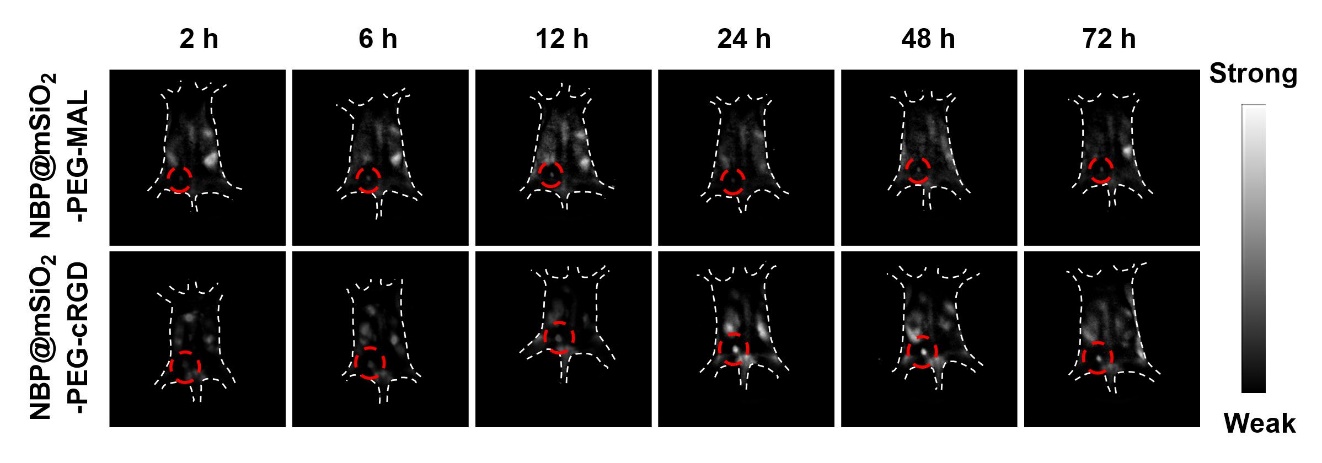


**Fig. S26.** *In vivo* fluorescence imaging of 4T1 tumor-bearing mice after intravenous injection of Cy5-labeled NBP@mSiO_2_-PEG-MAL (Cy5-NBP@mSiO_2_-PEG-MAL) or Cy5-labeled NBP@mSiO_2_-PEG-cRGD (Cy5-NBP@mSiO_2_-PEG-cRGD); The red circle represents the tumor.


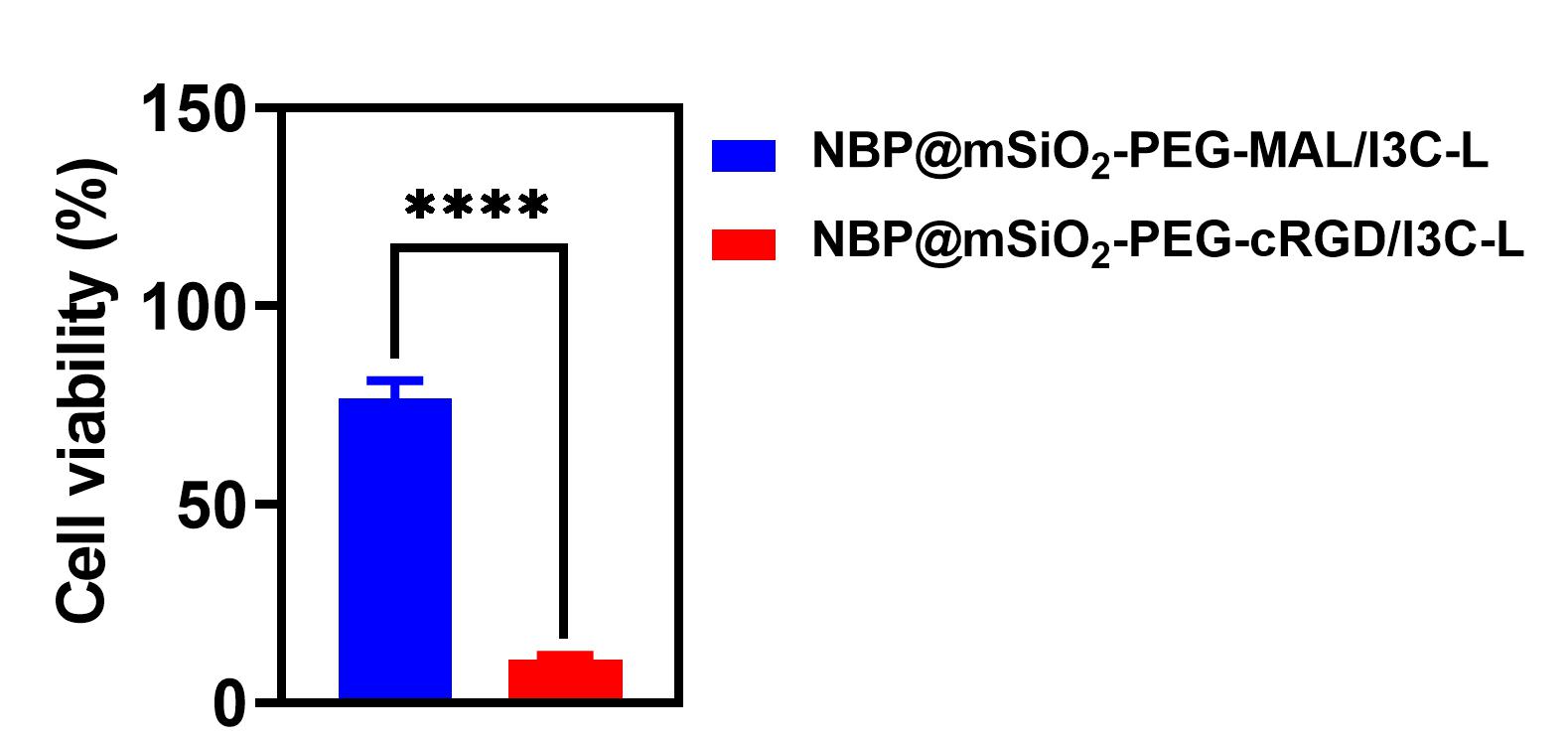


**Fig. S27.** Viabilities of 4T1 cells under different treatment scenarios (i.e., NBP@mSiO_2_-PEG-MAL/I3C-L and NBP@mSiO_2_-PEG-cRGD/I3C-L; “L” represents “808 nm laser irradiation at 2 W/cm^2^ for 10 min”). Data are expressed as the mean ± SD. Statistical analysis is performed using one-way ANOVA and Student’s *t* test. *****p* < 0.0001 represents a statistical significance.


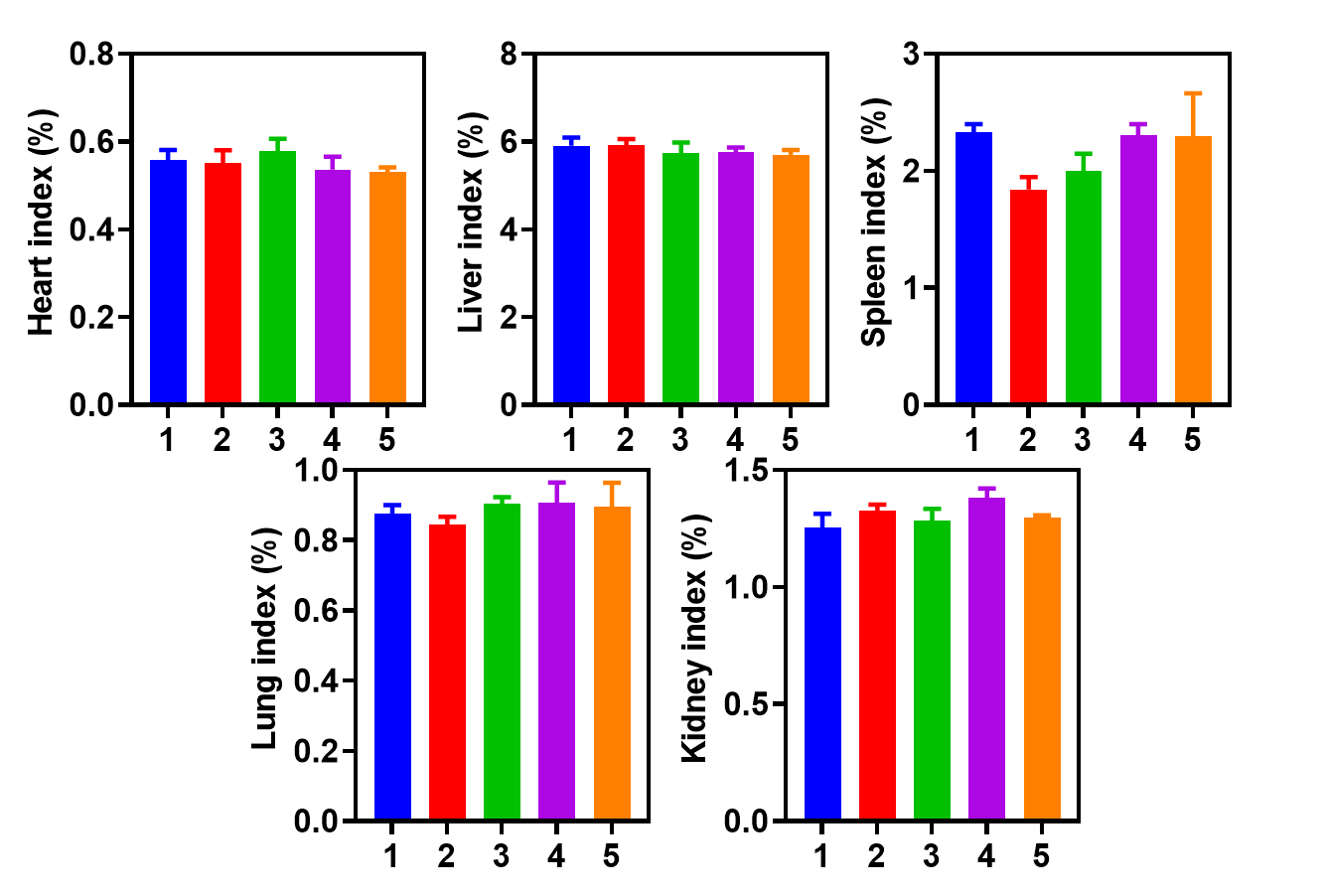


**Fig. S28.** Organ indices (i.e., heart index, liver index, spleen index, lung index, and kidney index) in mice after different treatments (1: PBS; 2: I3C; 3: NBP@mSiO_2_-PEG-cRGD/I3C; 4: NBP@mSiO_2_-PEG-cRGD-L; 5: NBP@mSiO_2_-PEG-cRGD/I3C-L; “L” represents laser irradiation).


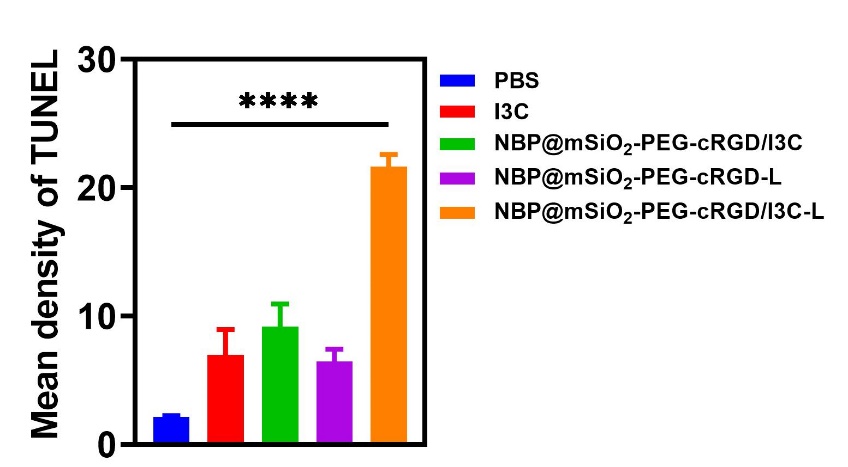


**Fig. S29.** Mean fluorescence intensity of TUNEL staining. *****p* < 0.0001 represents a statistical significance.


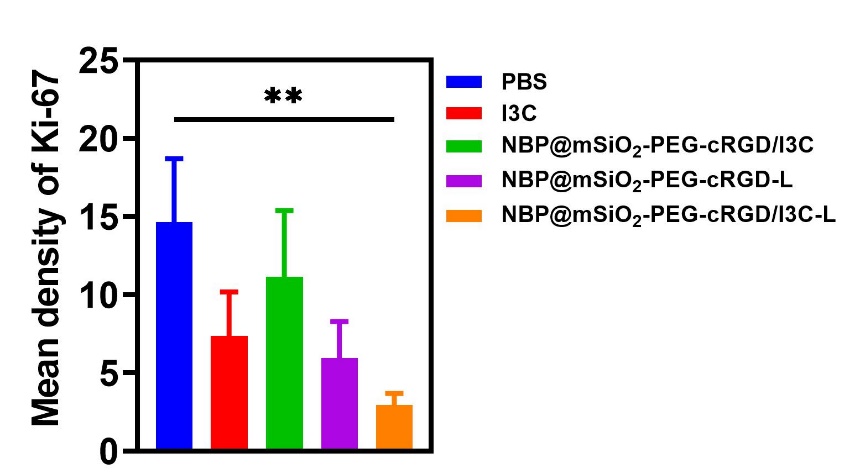


**Fig. S30.** Mean fluorescence intensity of Ki-67 staining. ***p* < 0.01 represents a statistical significance.


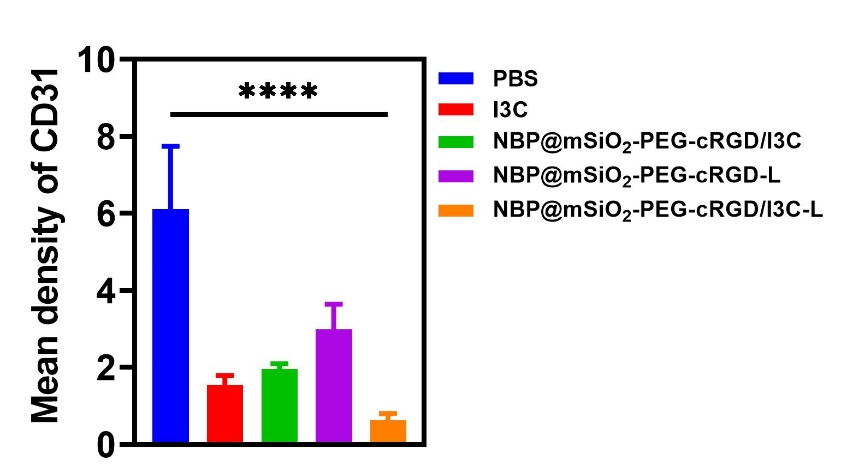


**Fig. S31.** Mean fluorescence intensity of CD31 staining. *****p* < 0.0001 represents a statistical significance.


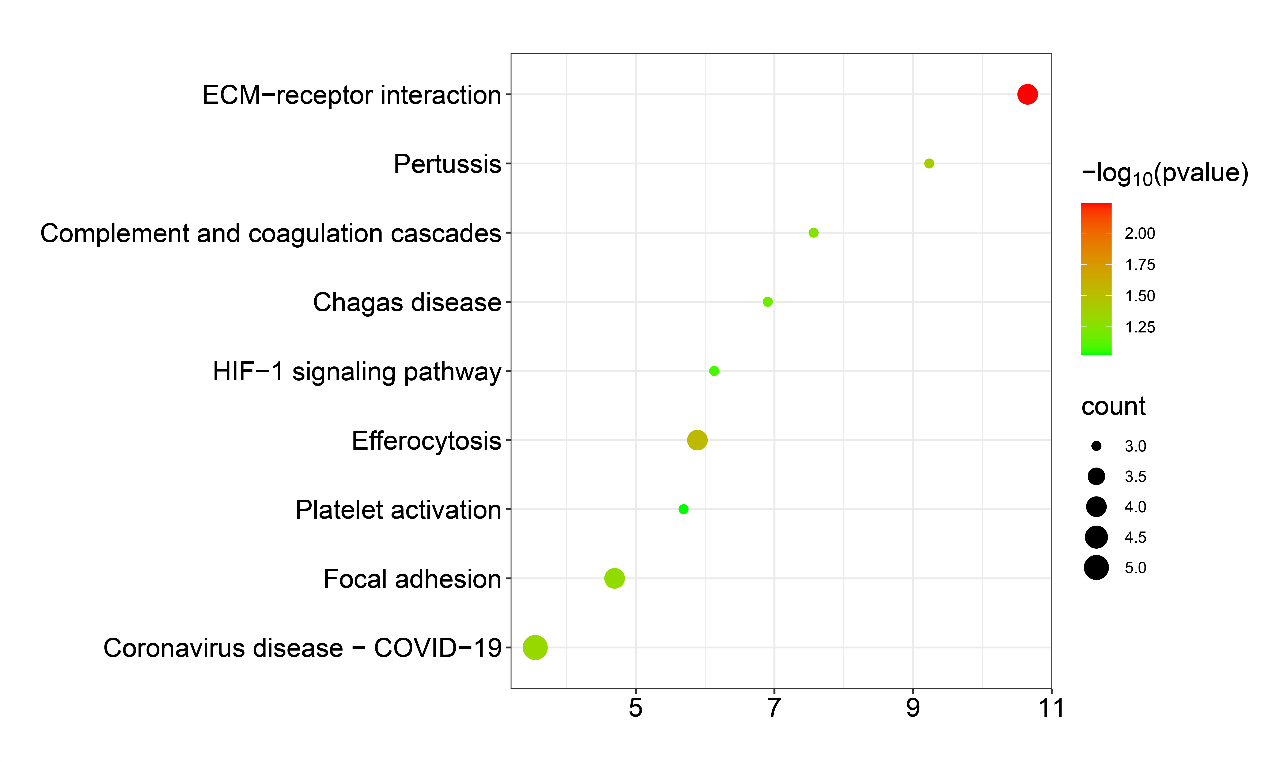


**Fig. S32.** KEGG enrichment analysis of down-regulated protein.
